# Mechanism of Membranous Tunnelling Nanotube Formation in Viral Genome Delivery

**DOI:** 10.1371/journal.pbio.1001667

**Published:** 2013-09-24

**Authors:** Bibiana Peralta, David Gil-Carton, Daniel Castaño-Díez, Aurelie Bertin, Claire Boulogne, Hanna M. Oksanen, Dennis H. Bamford, Nicola G. A. Abrescia

**Affiliations:** 1 Structural Biology Unit, CIC bioGUNE, CIBERehd, Derio, Spain; 2 Center for Cellular Imaging and Nano-Analitics (C-CINA) Biozentrum, University of Basel, Basel, Switzerland; 3 Institut de Biochimie et Biophysique Moléculaire et Cellulaire, Université de Paris–Sud, Orsay, France; 4 Institute of Biotechnology and Department of Biosciences, Viikki Biocenter, University of Helsinki, Finland; 5 IKERBASQUE, Basque Foundation for Science, Bilbao, Spain; Institut Pasteur, France

## Abstract

In internal membrane-containing viruses, a lipid vesicle enclosed by the icosahedral capsid protects the genome. It has been postulated that this internal membrane is the genome delivery device of the virus. Viruses built with this architectural principle infect hosts in all three domains of cellular life. Here, using a combination of electron microscopy techniques, we investigate bacteriophage PRD1, the best understood model for such viruses, to unveil the mechanism behind the genome translocation across the cell envelope. To deliver its double-stranded DNA, the icosahedral protein-rich virus membrane transforms into a tubular structure protruding from one of the 12 vertices of the capsid. We suggest that this viral nanotube exits from the same vertex used for DNA packaging, which is biochemically distinct from the other 11. The tube crosses the capsid through an aperture corresponding to the loss of the peripentonal P3 major capsid protein trimers, penton protein P31 and membrane protein P16. The remodeling of the internal viral membrane is nucleated by changes in osmolarity and loss of capsid-membrane interactions as consequence of the de-capping of the vertices. This engages the polymerization of the tail tube, which is structured by membrane-associated proteins. We have observed that the proteo-lipidic tube *in vivo* can pierce the gram-negative bacterial cell envelope allowing the viral genome to be shuttled to the host cell. The internal diameter of the tube allows one double-stranded DNA chain to be translocated. We conclude that the assembly principles of the viral tunneling nanotube take advantage of proteo-lipid interactions that confer to the tail tube elastic, mechanical and functional properties employed also in other protein-membrane systems.

## Abstract 2

Abrescia and colleagues demonstrate how the bacteriophage PRD1, a model membrane-containing virus, generates a self-polymerizing protein-lipid nanotube to deliver its viral genome to a host cell.

## Author Summary

Viral survival and propagation depend on the ability of the viruses to transfer their genetic material to a host cell. Viral genome delivery has been described for viruses that directly enclose their genome in a capsid or nucleocapsid, but not for internal membrane-containing viruses in which the genome is protected by a lipid vesicle enclosed by the icosahedral capsid. The latter infect organisms across the three domains of life. We use a range of electron microscopy techniques to reveal how one such virus, the bacteriophage PRD1, which uses gram negative bacteria as its host, delivers its double-stranded DNA to the bacteria across the cell envelope. The PRD1 bacteriophage is special in that it doesn't carry a rigid tail; rather it creates a tube tail when needed at the time of infection to pass its DNA through to the host. We now show that this tube formation is accomplished via concerted restructuring of the icosahedral capsid and remodeling of the internal icosahedral protein-rich virus membrane. We also find that this tail tube is studded with membrane-associated proteins and its internal diameter allows one double-stranded DNA chain to be injected. Finally, we capture PRD1 in 3-D with the proteo-lipidic tail piercing the gram-negative bacterial cell and shuttling its viral genome *in vivo*. These results provide insights into a new mechanism of viral genome delivery.

## Introduction

A fundamental step in the lifecycle of all known viruses is the genome translocation into the target cell. Although this process has been elucidated for several enveloped viruses [1],[2], and for double-stranded (ds) DNA head-tailed bacteriophages [3]–[7], equivalent information on internal membrane-containing viruses is scarce.

**Figure 1:**
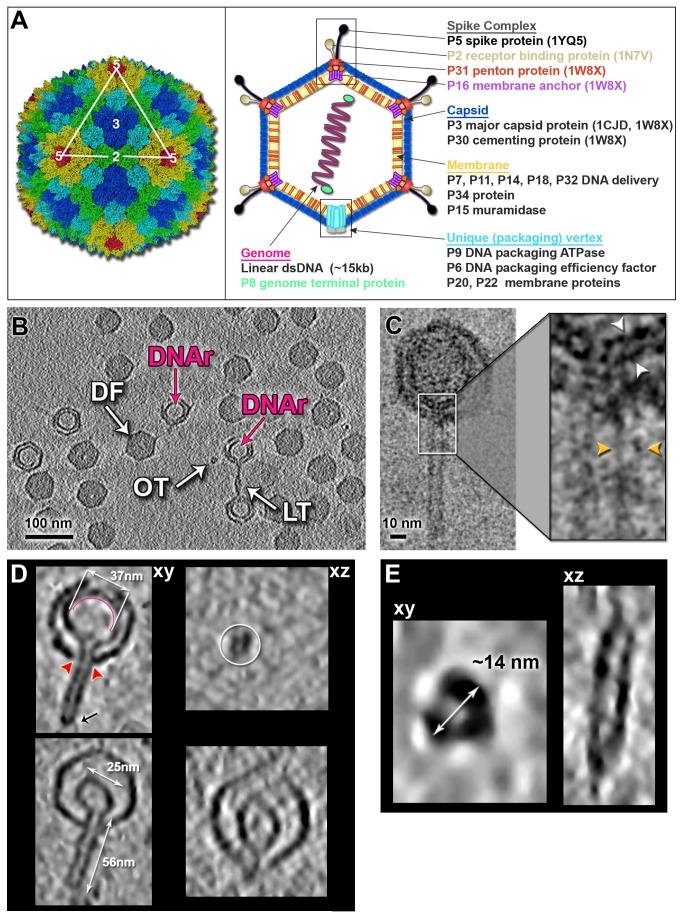
Cryo-ET structures of individual wt PRD1 with a tail tube. (A, Left) PRD1 capsid organization represented as in [8] with each of the 4 P3 MCPs trimers composing the asymmetric unit coloured in green, cyan, blue, and yellow; in red is the vertices penton protein P31 (PDB ID 1W8X). The white triangle marks a virus facet, and 2, 3, and 5, respectively, the 2-, 3-, and 5-fold icosahedral symmetry axes. (A, Right) Schematic presentation of wt PRD1 architecture according to the current knowledge (numbers in parentheses identify the corresponding protein structures in the Protein Data Bank). (B) Overall view of a section of a reconstructed tomogram with the different wt PRD1 related structures (DF, DNA-filled particle; OT, orthogonal tube; LT, longitudinal tube); some semi-empty particles display a darker region within the membrane indicating residual DNA (DNAr). (C) Cryo-image of PRD1 with a tube at 50,000× magnification (2 µm under focus) showing the bilayer structure (inset) of the vesicle (white arrowheads) and of the tube wall (gold arrowheads). (D, Top Left) Tomographic central *xy* section of a representative wt PRD1 particle with a tube possibly at initial stage of DNA ejection; red arrowheads indicate the vertex aperture from where the tube protrudes and the black arrow the conical tip of the tube. A semicircular red line within the vesicle marks the presence of residual genome. (D, Top Right) An orthogonal view (*xz*) of the tube. (D, Bottom Left and Right) As corresponding top but with a wt PRD1 particle with a tube possibly at the final stages of the DNA ejection with the vesicle showing a “map pin” morphology. (E) One individual tube with the long axis (quasi-)orthogonal to the tilting plane. (E, Left) Density pattern of the tube from a central tomographic section (*xy*) and (E, Right) *xz* section of the tube. White double head-arrows mark the dimensions of the different particle structural elements; tomograms were denoised using TOMOAND [58] and displayed in AMIRA (Visage Imaging GmbH, Berlin).

PRD1 is an internal membrane-containing dsDNA bacteriophage (family *Tectiviridae*) whose crystallographic studies (Figure 1A, left) have provided unprecedented insights into the assembly mechanism of large complex viruses [8]–[10]. With several other such examples [11]–[16], this has led to a novel principle of classifying viruses based on their major capsid protein (MCP) fold [17],[18]. Such viruses pose fundamental questions about the morphogenesis of the membrane and the genome packaging and ejection processes that utilize the lipid bilayer enclosing the genome.

PRD1 packages its genome capped with terminal protein P8 [19] into a preformed membrane-containing procapsid using the packaging complex at the unique vertex specifically composed of (*i*) the packaging ATPase P9, (*ii*) the packaging accessory protein P6, and (*iii*) two small membrane proteins P20 and P22 (Figure 1A, right) [19]–[22]. The other 11 vertices are different, being constructed from (*i*) the vertex-stabilizing membrane protein P16, (*ii*) penton protein P31, (*iii*) spike protein P5, and (*iv*) receptor binding protein P2 (Figure 1A, right) [8],[23]–[25]. So far, the 3D structure of this unique vertex is not known.

Interestingly, PBCV-1, an algae infecting virus, and the largest virus described, Mimivirus infecting *Acanthamoeba polyphaga*, both with an internal membrane and thought to belong to the same structure-based virus lineage with PRD1, have also recently been shown to possess a unique vertex [26]–[29].

Activation of the PRD1 infection process in a broad range of gram-negative hosts such as *Escherichia coli*, *Salmonella enterica*, and *Pseudomonas aeruginosa* is triggered by specific binding of the viral protein P2 to the cellular receptor. A model for DNA delivery has been postulated from biochemical and genetic studies which point to the involvement of viral membrane proteins P7/P14, P18, and P32 in the formation of the putative membranous tail tube protruding from one vertex [30],[31].

Here, we combine immuno-labelling, cryo-electron microscopy, and cryo-electron tomography (cryo-ET; single-particle, subtomogram averaging, and cellular tomography) analyses using wild-type (wt) PRD1, DNA-packaging-deficient mutant Sus1 particles (i.e., procapsids) and *in vivo* analysis of virus-infected cells to report a new principle of virus–cell interaction essential for viral genome translocation.

We suggest that the PRD1 tube protrudes from the same unique vertex used for DNA packaging and that it is structured, implying the direct involvement of self-assembling and lattice forming membrane-associated proteins. Furthermore, we demonstrate that the internal vesicle in DNA-less procapsids can undergo acrobatics. This ability underscores the general elasto-mechanical properties of giant unilamellar (proteo-)vesicles and the triggering factors of the viral vesicle shape transition leading to the assembly of the tail tube. This assembly process and the tunnelling of the viral tail tube through the cell envelope is reminiscent of cellular membrane nanotubes used in cell-to-cell communication [32],[33].

## Results

### DNA Packaging and Ejection: A “Code-Sharing” Usage of the Same Vertex

The samples used throughout this study contained a mixture of intact viruses and empty particles as well as particles with a tube and individual tubes devoid of capsid (Figure 1B). In 2D cryo-images of PRD1 tail tube at 50,000× magnification, the tube's wall appeared as a bilayer structure with dimensions similar to those of the vesicle within the capsid (Figure 1C). We adopted ageing of the PRD1 sample as a way to obtain particles with a tube because a trigger for synchronous genome ejection *in vitro* is as yet unknown. Whether the genome ejection machinery uses the same vertex through which the genome is packaged remains unclear. Circumstantial evidence based on immuno-labelling of packaging-vertex-associating proteins P20 and P6 suggested that the DNA packaging and ejection vertices might coincide, implying that upon tube formation P20 and P6 may be dislocated (Protocol S1 and Figure S1). We also performed subtomogram averaging of intact wt PRD1 particles with no imposition of icosahedral symmetry and with a loose shell mask that included the vesicle in the alignment process in the attempt to detect coarse asymmetrical structural features. Although we confirmed previous results on the conformational flexibility of the spike complex [23], at this level of analysis we could not firmly detect structural differences between the 12 vertices [5.8 nm resolution at the 0.5 threshold of the Fourier shell correlation (FSC); Figure S2A]. Further classification and higher resolution studies are required to fully resolve the structure of the packaging vertex.

### Structural Transition of the Internal Membrane from Icosahedral to Tubular

**Figure 2:**
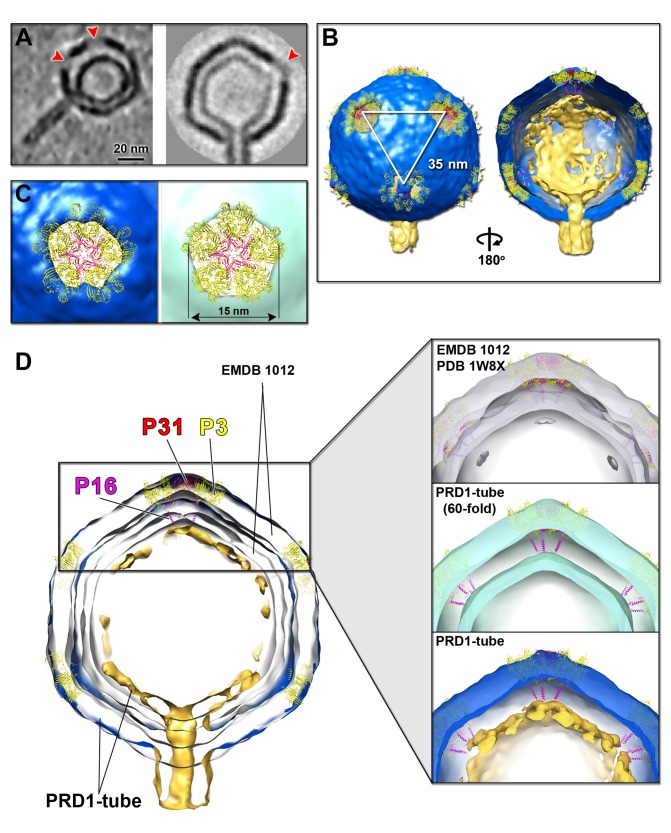
Apertures at the 5-fold vertices in wt PRD1 capsid. (A, Left) Central tomographic section of a wt PRD1 particle with a tube showing two contiguous de-capped vertices (red arrowheads) and nearby the shrinking of the vesicle volume within the capsid (denoised as in Figure 1D). (A, Right) Central section of the subtomogram averaged wt PRD1 capsid displaying one of the de-capped vertices (red arrowhead); not to scale with previous. (B, Left) Isosurface representation of the nonicosahedrally symmetrised subtomogram averaged PRD1 with tube volume (capsid blue, membrane/tube gold; displayed at 1.5σ in Chimera [59]) with the atomic models of the vertex complexes, the peripentonal P3 MCPs trimers (yellow), the P31 penton protein (red), and the membrane protein P16 with its transmembrane helices (magenta) (PDB code 1W8X) at the three contiguous de-capped vertices joined by white lines (vertex-to-vertex ∼35 nm). (B, Right) Cut-through of the PRD1 particle isosurface (rotate by 180° from left) showing the missing density of the vesicle regions proximal to the de-capped vertices (contoured at 0.35σ). Hereafter, the boundaries between capsid shell, vesicle, and tube were assigned by eye and prior knowledge [8]; the structural analysis of the PRD1 atomic model in the context of our subtomogram averaged maps was carried out as described in Material and Methods. (C, Left) Enlarged view of one of the de-capped vertices (contoured at 1.1σ) whose aperture matches the loss of the spike complex; (C, Right) As left but subtomogram averaged PRD1 tube density with 60-fold symmetry imposed (pale-green, contoured at 2.5σ). (D, Left) Cut-through of the nonicosahedrally symmetrised PRD1 tube density (displayed at 0.5σ; capsid in blue, vesicle and tube in gold) superimposed onto the PRD1 reference model (semitransparent light-grey density contoured at 1.2σ with labelled atomic models of P3, P31, and P16 coloured as in B) showing the size agreement of the corresponding capsid shells but not of the vesicle. (D, Inset) Enlarged views showing the spatial relationship between the peripentonal P3 MCPs trimers, P31 pentons, and P16 proteins relative to the vesicle densities, respectively, of PRD1 icosahedral cryo-EM map (Top), of 60-fold symmetrised averaged cryo-ET map (semitransparent pale-green) (centre) and of nonicosahedrally symmetrised PRD1 tube density (bottom); in the latter two cases it is apparent the loss of P16 membrane interactions with the vesicle.

We focused our analysis on 3D tomographic reconstructions of individual PRD1 particles with a tube on which icosahedral symmetry was not imposed (Figure 1D). These tomographic reconstructions showed that the icosahedral capsid was largely preserved, with a unique tube with a diameter of ∼14 nm and with variable lengths (mean 51.4±9.4 nm, *n* = 70) protruding from a single vertex (Figure 1D–E). In rare cases the distal part of the tube appeared closed (black arrow in Figure 1D, top left). As far as can be judged in the presence of the missing-wedge effect, the capsid is not structurally compromised except at some vertices, including the one with the protruding tube, where an opening of ∼15 nm diameter allows the tube to exit through the vertex (Figures 1D and 2A, left). Overall capsid and membrane thicknesses agree with those reported from previous PRD1 studies (Figure 2) [8],[9],[34]. Interestingly, subtomogram averaging of the capsids with a tube carried out using *Dynamo* software (at 6.4 nm resolution, no icosahedral symmetry imposed; Figures 2A, right and S2B, top left) [35] suggests that there is preferential de-capping of contiguous vertices, one of which is adjacent to the vertex from which the tube protrudes (Figure 2B). These apertures imply the loss of the peripentonal MCPs (P3), membrane proteins P16, penton proteins P31, and vertex-associating proteins P2 and P5 (Figure 2C). In turn, this de-capping of the vertices leads to the loss of the P16 protein interactions and P3 N-terminal contacts with the underlying membrane (Figure 2D).

Whereas in the virions the membrane follows icosahedral symmetry [8],[9],[34], distinct vesicle morphologies were detected in the individual tomograms of the particles with a tail tube. These ranged from a membrane not fully deformed, most probably illustrating the initial stages of DNA ejection (Figure 1D, top), to particles where the membrane appears to clearly deflate in proximity of the de-capped vertex complexes (Figures 1D, bottom, and 2A, left). In some tomograms, clear density attributable to DNA was also visible within the vesicles with a protruding tube (Figure 1B and 1D, top left).

In addition, particles where the vesicle shape was drastically compromised resembling a “map pin” were seen (Figure 1D, bottom). The change in the vesicle size from an icosahedral one to a membrane with a protruding tail tube (as shown in Figure 1D, below) causes a drastic reduction in membrane area (∼30%) and volume (∼60%), reflecting one of the last stages in DNA translocation. Additionally, the exit direction of the tail tube was not always aligned with the icosahedral 5-fold axis but angled (Figure 1D, bottom), in some cases, with a deflection of ∼20° (Protocol S2).

### PRD1 Procapsids Devoid of the Genome Can Form Nanotubes

**Figure 3:**
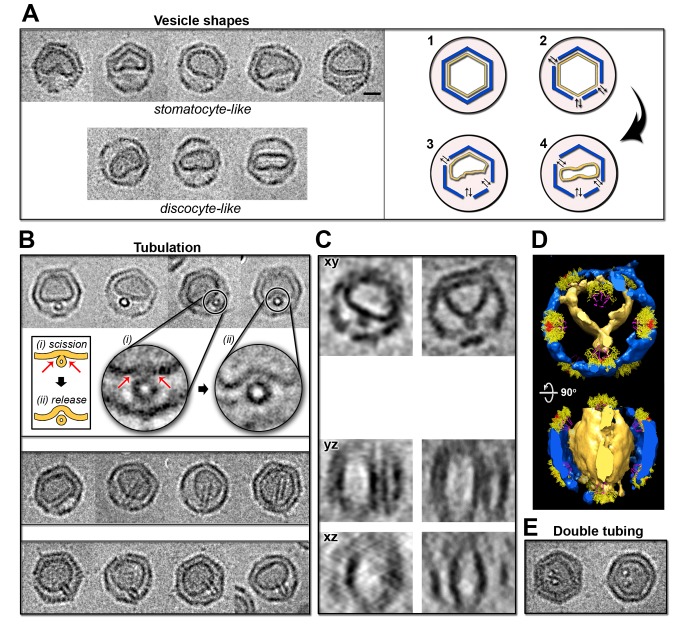
Vesicle morphology in PRD1 procapsid. (A, Left) Gallery of 2D cryo-images of vesicle shapes (top, stomatocyte-like; bottom, discocyte-like) within the PRD1 procapsids; scale bar, 20 nm. (A, Right) A cartoon of the steps leading to vesicle transformation: (1) capsid is intact and vesicle not exposed to environment, (2) opening of one or more vertex complex (by *in vitro* or *in vivo* triggering) destabilizes the vertex–vesicle interactions and directly exposes the vesicle to surrounding conditions, and this induces the shape transitions (3 and 4). (B) Gallery of 2D cryo-images of PRD1 procapsids (scaled to A, Left) with a tube with characteristic views. (B, Top) Particles with forming tube viewed along the long axis of the tube with insets highlighting two possible tubulation stages: (*i*) scission (red arrows indicate the contact points to the vesicle) and (*ii*) tube release; both stages are schematically represented by the cartoon on the left. (B, Centre) Particles with a tube viewed orthogonal to the long axis of the tube. (B, Bottom) Additional views of particles with a tube. (C) *xy*, *yz*, and *xz* sections of two different PRD1 procapsids cryo-ET reconstructions (side-by-side) unequivocally showing that the budding of the tube occurs within the capsid and tangentially to the viral vesicle and recapitulating the views of 2D cryo-images in (B). (D, Top) Isosurface representation of the 3D volume of the procapsid shown in (C, Right) with docked the 12 vertex complexes (as Figure 2B and 2C) viewed along the icosahedral 2-fold axis and 90° rotated (D, Bottom). (E) 2D images of procapsids with a membrane with two budding tubes viewed along their long axis (scaled to A, Left).

**Figure 4:**
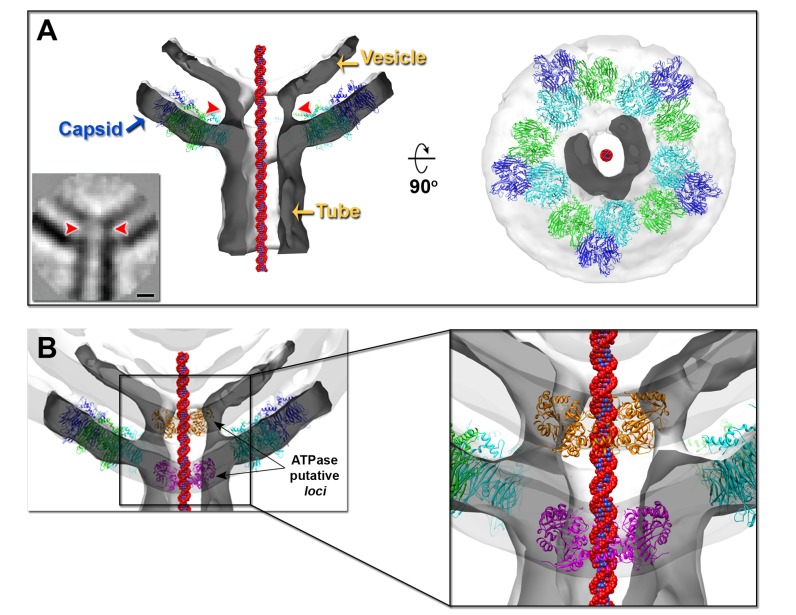
Subtomogram averaging of the PRD1 DNA exit gate. (A, Left) Cut-through of the isosurface of the subtomogram averaged cryo-ET volume of the PRD1 DNA exit gate (semitransparent, white-smoke; clipped surface in dark-grey contoured at 1.1σ) with superimposed MCP P3 trimers (represented in cartoon and color-coded as in Figure 1A, left) next to the de-capped vertex and with a space-filled model of a B-DNA (red and blue) shown within the cavity of the tube. (A, Bottom Left) The corresponding central section (0.88 nm thickness) with the red arrowheads pointing at the density at the interspace between the vesicle and the capsid, which is linearly connecting the vesicle and the tube (scale bar, 5 nm). (A, Right) Cut-through view of the density corresponding to the external tube stemming from the de-capped vertex (darker grey area). (B) Superimposition of the exit gate reconstruction (white smoke and dark-grey) onto the PRD1 cryo-EM density (semitransparent light-grey; contoured at 0.9σ) with hexameric models of the first viral ATPase of a nontailed virus (represented as cartoon in magenta and orange) [36] manually fitted in two putative loci along the tube density indicating the mismatch in size between the diameters of tube and the putative hexameric ATPase as highlighted in the inset.

In previous studies, the membrane isolated from empty PRD1 procapsids (*sus1* mutant) by guanidine hydrochloride treatment has been shown to form tubular structures, whereas the DNA-containing vesicles isolated from the virions mainly adopt a spherical shape [31]. Mutant *sus1* has a defect in the packaging ATPase gene *IX* (encoding protein P9), and thus it does not package DNA. Using a similar ageing regime and buffer conditions to that used for wt PRD1, we inspected the ability of the procapsid to form the tail tube and the procapsid membrane morphology by 2D and cryo-ET imaging. Indeed, tubes were assembled and projected from one of the vertices as for wt PRD1 (Figure S3). Remarkably, the membrane in the procapsid exhibited far more varied morphologies than the membrane in the virion. These diverse membrane shapes within the capsid included stomatocyte-like, discocyte-like shapes, and internal tubes budding and pinching off as extra vesicle from the larger one (Figure 3A–B). Intriguingly, these tails tubulate and pinch off tangentially to the vesicle (Figure 3B–D). This direction of tubulation is completely different from that observed in other PRD1-tube particles in which the tube polymerizes orthogonally to the vesicle (Figures 1D, 4, and S3). In addition, two tubes were occasionally visible budding from the vesicle (Figure 3E).

### Absence of Any Ordered Multimeric Structure at the Exit-Portal of PRD1 Tail Tube

To grasp whether a conserved structure was present at the exit-portal between the vesicle and the capsid aperture (hereafter called “gate”), several subregions corresponding to the gate (*n* = 138) were averaged (Figure 4A). Manual fitting of a hexameric model of the only viral packaging ATPase structure available (STIV2 protein B204, [36]) from a nontailed dsDNA virus into the PRD1 gate structure indicates a size mismatch between the tube and the multimeric ring (Figure 4B). Also, within the current resolution limits (∼5.7 nm; Figure S2B, top right), there were no indications of an ordered multimeric ring-like structure of radius >8 nm at the gate (red arrowheads in Figure 4A, left) that could correspond to a portal or to a larger multimer of the packaging ATPase P9 or the packaging efficiency factor P6 or their heteromultimer.

Notably, the density corresponding to the vesicle forms a continuum with the tube, resembling a funnel with the narrow end traversing the PRD1 open vertex (Figure 4A, left). Cutting the averaged volume nearby this aperture exposes the density of the tube appearing to stem rather like a hoof-shaped object (Figure 4A, right).

### The Viral Membranous Tube Arranges as an Ordered Structure During Its Assembly

**Figure 5:**
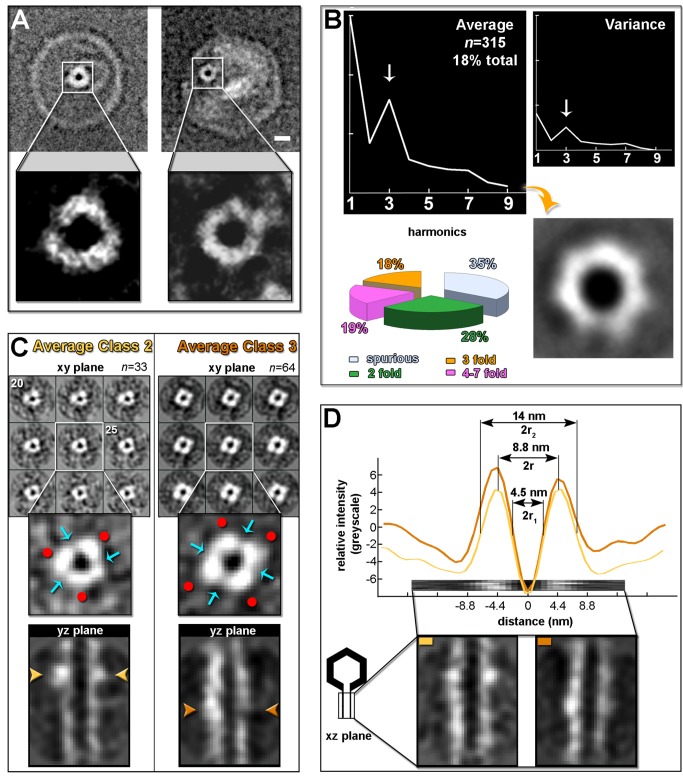
Rotational analysis of the tube and tube morphology. (A) Examples of a cryo-image of wt PRD1 (∼6 µm under focus) (Left) and a negative stain image of PRD1 procapsid (∼4 µm under focus) (Right) with orthogonal views of the tube. The insets (4× magnification) highlight the density pattern of the orthogonal 2D projection of the tube. The density of the phage across the figure has been inverted (white) for clarity. Scale bar, 10 nm. (B, Top Left) Plot of the averaged rotational spectra corresponding to the subpopulations for which the 3-fold harmonic component was dominant with correspondent variance (Top Right) and resulting 2D averaged density pattern (Bottom Right) obtained by the KerdenSom analysis of 1,758 rotational spectra of 2D cryo-images of orthogonal tubes boxed out from PRD1 procapsids. (B, Bottom, Left) A pie-diagram summarising the distribution of rotational symmetries (see also Figure S4). (C, Top Left) Consecutive representative *z*-slices from left to right starting at slice 20 (0.88 nm thickness) of the tube volume obtained by averaging 33 subtomograms (average class 2) (white, density pattern). (C, Top Right) As left but averaged tube obtained from 64 subtomograms (average class 3). (C, Insets) Central cross-sectional density pattern with high- and low-contrast regions marked respectively by a red dot and a cyan arrow. (C, Bottom) Central *yz* section corresponding to the tube average maps with gold and dark-gold arrowheads pointing at the ring-like structure (see also Figures 7 and S5). Both averaged volumes were low-pass band filtered at 5.0 nm before displaying. (D) Mean density profiles along *z* (Top) calculated from the central *xz* section of the class 2 (gold line) and class 3 (dark-gold line) averaged tubes (Bottom). Dimensions of the tube parameterized as hollow cylinder (r_1_, inner radius; r_2_, outer radius; r, average radius) have been given for the averaged tube 2.

Analysis of 2D images and subsequent 3D tomographic reconstruction of individual PRD1 particles with a tube showed a limited number of particles with the long axis of the tube aligned (or *quasi*) with the direction of the electron beam (hereafter called “orthogonal” tubes) (Figure 1B). This orientation in 2D images of PRD1 suggested that the tube might possess rotational symmetry (Figure 5A). Conversely, previous Fourier analysis of individual tubes with the long axis lying quasi-parallel to the imaging plane (hereafter called “longitudinal” tubes) showed too weak periodicity to unequivocally support a helical symmetry for the tube.

Thus, we performed rotational symmetry and classification analyses on a set of orthogonal tubes (*n* = 1,758) extracted from 2D cryo-images of PRD1 procapsids (see Material and Methods). These analyses suggested a subset of tubes with a 3-fold rotational symmetry (18%) besides those displaying 2-fold symmetry (28%), spurious symmetries (35%), or higher symmetries (together summing up to 19%) (Figures 5B and S4). Such a distribution of rotational symmetries prompted the possibility that (*i*) we were framing tubes at different stages of assembly (or disassembly) with structural heterogeneity, (*ii*) we were not viewing the tubes exactly along their symmetry axis, and/or (*iii*) the structure of the tube possessed also a helical component. Therefore, we extended this study to reconstructed cryo-electron tomograms of wt PRD1 particles with a tube (also showing the preferential longitudinal orientation; Figure 1C). Due to the well-known missing wedge effect on cylindrical objects in this orientation, we performed subtomogram averaging of the tubes to increase signal-to-noise, to compensate for the missing-wedge loss, and to reliably assess the presence of a density pattern. However, no symmetry was assumed or imposed during this process. Prior to averaging, 167 subboxed tubes (with the axes oriented along the *z*-direction) were classified using a multireference alignment approach with four references (see Material and Methods, Protocol S2, and Figure S5A). Of the four resulting classes (class 1, *n* = 35; class 2, *n* = 33; class 3, *n* = 64; class 4, *n* = 35), based on relatively higher mean cross-correlation (cc) with the corresponding references, reasonable angular distribution of the set of tubes covering the geometric sphere and visual inspection of the densities, only class 2 and class 3 tube averages were further considered [class 1 recapitulated basic structural features of class 2 and 3 (respectively cc_1–2_ = 0.61 and c_1–3_ = 0.69), whereas class 4 was the most structurally incongruous (cc_4–2_ = 0.49 and cc_4–3_ = 0.53; Figure S5B)]. The corresponding *z*-slices showed a pattern of alternating strong (red dots) and weak (cyan arrows) densities (Figure 5C, top and insets) that we interpreted as the cross-sectional views of upright strands (stronger densities) skeletonizing the tail tube and interacting laterally to each other. This pattern was not fixed across the *z*-slices (see, e.g., *xy*-slice 20 *versus xy*-slice 25 in Figure 5C, left top). To assess whether this lobular distribution of density was artifactual, simulation of tomographic data using a featureless cylindrical shell supported the *bona fide* averaged reconstructed tube models (see Protocol S3 and Figure S5D). Furthermore, both averaged volumes contained an additional unique ring-like structure (Ø∼18 nm) crowning the tube, although it was much more distinctive in class 2 (gold arrows in Figure 5C, bottom; this ring was not present in the averaged volume of class 1; Figure S5B). Thus, both 2D image and 3D volume analyses indicated that the tube possesses a degree of order and structure. However, the morphological variability noted in individually visualized PRD1 particles with a tube (Figures 1B–D, 2A, left, and 3), in the distribution of rotational symmetries (Figure 5B), as well as in the resulting averaged tube volumes (Figures 5C and S5B) imply that the structure of the tube is variable. The parameters of the two averaged tube models calculated from the mean density profile along *z* of the central *xz* section indicate that both tubes possess an equivalent inner diameter (2r_1_∼4.5 nm) but possibly slightly different outer diameters (the smallest being 2r_2_∼14 nm) (Figure 5D).

### In Vivo DNA Translocation During Virus Genome Delivery

**Figure 6:**
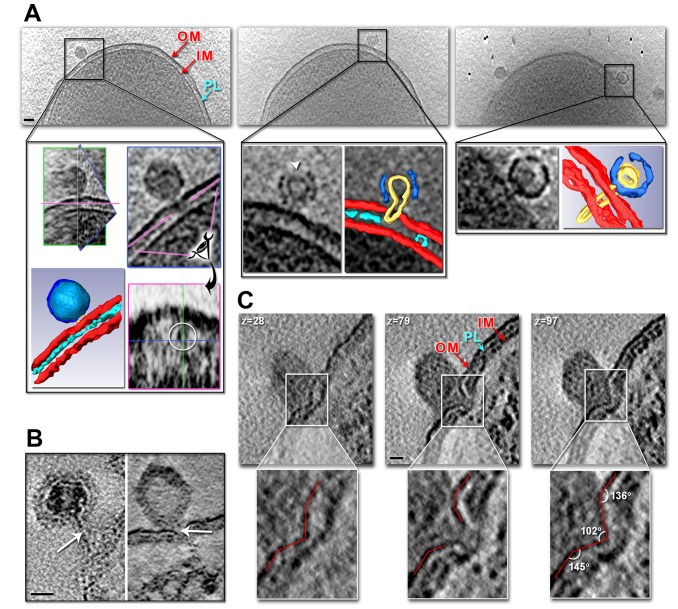
*In vivo* PRD1 genome delivery. (A) Tomographic slices of an *Escherichia coli* cell infected by wt PRD1 (∼30 min p.i.). Scale bar, 50 nm. (A, Left) Full particle, outlined in a black line, with forming tube crossing the outer membrane (OM). Inner plasma membrane (IM) and peptidoglycan layer (PL) are indicated. (A, Inset, Top Left) Particle viewed through three intersecting planes marked in green, blue, and magenta with corresponding blue and magenta planes on the right. (A, Inset, Below Left) A segmentation of the particle colour coded as Figure 2B, left with superimposed density derived from the atomic model (PDB code 1W8X) in light-blue, in red the outer and inner membranes, and cyan the peptidoglycan layer. (A, Centre) Semi-full particle, outlined in a black line. (A, Centre, Inset) A tomographic slice (Left) with superimposed segmentation of the capsid and cell envelope (Right). The white arrowhead indicates additional openings of the capsid. (A, Right) An almost empty particle, outlined in a black line. (A, Right, Inset) Tomographic slice showing a particle with a “map-pin” shape vesicle and full-length tube penetrating the cell envelope (Left) and corresponding segmentation (Right). (B) Tomographic slices of epon sections showing a DNA-filled wt PRD1 particle with tube far away from cell (Left; 5 min p.i.) and a DNA-devoid wt PRD1 with the tube closer to the cell (Right; 30 min p.i.) both with DNA visible as a darker linear density within the tube as marked by the white arrows; scale bar, 20 nm. (C) Sequential tomographic *z*-slices (top left corner) of a wt PRD1 particle (epon embedding; slice thickness 0.44 nm; 30 min p.i.) proximal to the outer membrane and inducing membrane deformation (see also Movie S2). Scale bar, 20 nm. Red lines in the insets below outline the drastic changes in cellular membrane morphology as seen above.

PRD1 genome entry occurs in a few minutes, inducing superinfection immunity [37]. This does not prevent other viruses binding to the cell but blocks the entry at a later stage, allowing entry intermediates to be detected. To visualize the PRD1 DNA delivery through the membranous tail tube *in vivo*, we used cellular cryo-tomography and tomography analysis on *S. enterica* and *E. coli* infected with a high multiplicity of infection (MOI = 30). For cellular cryo-tomography, whole infected *E. coli* cells were vitrified ∼30 min postinfection (p.i.). From six tomograms 11 viruses were analysed, revealing nine tail tubes with a diameter 15.9±1.7 nm. Viruses were visualized at distinct stages of the infection process—for example, (*i*) a DNA-containing particle with the tail tube piercing the outer membrane (Figure 6A, left), (*ii*) a half-empty particle (Figure 6A, centre), and (*iii*) an empty particle with a deformed vesicle morphology within the capsid (Figure 6A, right).

For cellular tomography, the infection process in *S. enterica* was analysed at 5 and 30 min p.i. (Movie S1). At 5 min p.i., based on 43 tomograms, most of the viruses (*n* = 119 in total) attached to the cell were still full of DNA. In 92 cases, tubes could be clearly visualized with a diameter 14.3±5 nm. Some capsids were seen to adhere to the cell outer membrane, whereas in others the capsids were found separated from the cell surface, having a part of their tubes standing outside the outer membrane (Figure 6B). In the latter case, the distance between surfaces of the bacterial outer membrane and the virus capsid varied from 5 to 44 nm, with an average of 19.3 nm (*n* = 21). When the entire tail tube was visible upon cell envelope penetration (for 13 viruses), its length was 47.6±4.5 nm. In some cases, DNA injected from the virus capsid could be seen as a central linear density within the tail tubes (Figure 6B).

At 30 min p.i., viruses (53 viruses extracted from 29 tomograms) appeared empty with no visible dense material inside the capsid (Figure 6B, right), thus indicating that they had most likely injected their genetic material. The tubes had a diameter of 13±7 nm (*n* = 36) and a length of 36±15 nm (*n* = 32). The distance from the bacterial outer membrane and the virus capsid varied from 10 to 24 nm, with an average of 15 nm (*n* = 20). Occasionally, a clear invagination of the inner and outer host membranes was visualized where the incipient tube pinched the cell envelope (Figure 6C and Movie S2).

## Discussion

### Dynamics of the Viral Membrane

**Figure 7:**
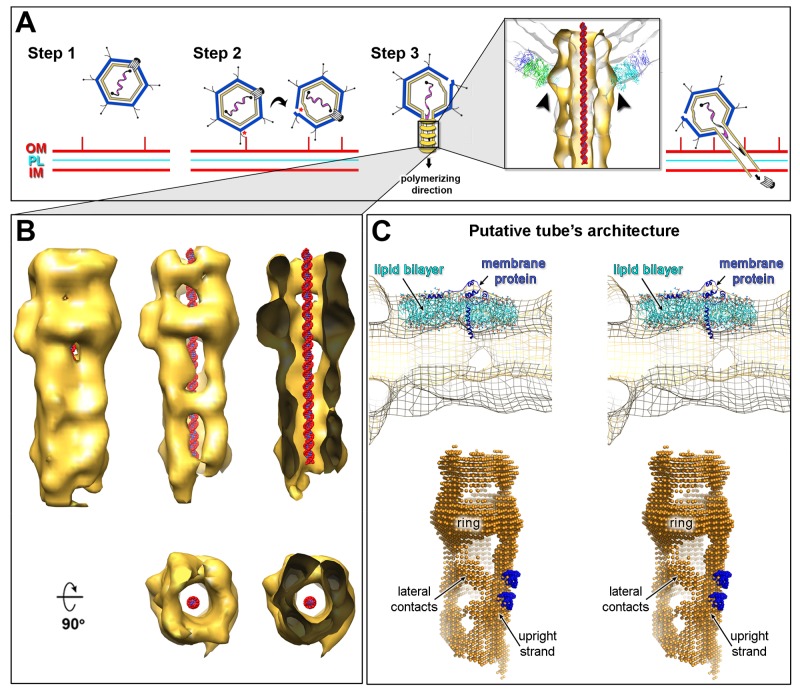
Schematics of PRD1 genome translocation via tunnelling nanotube and model of PRD1 membranous tail tube structure. (A) Sequential steps of PRD1 infection and tube formation. Step 1, PRD1 particles approach the cell surface in a random orientation. Step 2, after binding to the cellular receptor, partial rolling/tumbling of the particle due to solvent movement and/or binding of further viral protein P2 or secondary binders such as P5 proteins to cellular receptors guide the re-orientation of the unique vertex almost orthogonal to the cell. De-capping of the cell-interacting vertex triggers the membrane reshaping as a consequence of the loss of vertex-stabilizing protein P16 interactions and the influx of components of the extracellular milieu through the opened vertex (cf., change in osmolarity). Step 3, the vesicle transformation leads to the tube polymerization and piercing of the cell envelope as well as digestion of the peptidoglycan layer by virion-associated enzymes [30]. Once in the cytoplasm, the tip of the tube unplugs and the DNA is translocated relative to the tube from the vesicle into the cytoplasm. (A, Inset) Matching of the ring-like structure (black arrowheads) within the capsid region of the gate volume (white-smoke). (B, Left) Isosurface representation of the averaged model tube 2 (*n* = 33, Figure 5C, Left) with the distinctive ring-like structure nearby the top end of tube (low-pass band filtered to 5.0 nm and contoured at 0.8σ; gold) with a model of B-DNA fragment manually docked within the channel viewed perpendicular to the tube long axis. (B, Centre) As left but displayed at higher contouring level (1.4σ) (Top) and 90° rotated (Bottom). (B, Right) As centre but viewed through a cutting-plane halfway into the tube (Top) and with a 90° rotated view of the tube cut-through above the tip-end (Bottom). For a superimposition of the averaged density of tube 2 onto average tube 3, see Figure S6. (C) Putative tube's architecture. (C, Top) Stereo-view of a portion of the averaged tube 2 density (as mesh contoured at 1.4σ; 90° rotated relative to B, Top) cut-through its longitudinal axis with manually fitted a lipid bilayer model (cyan) and a membrane protein (blue). (C, Bottom) Stereo-view of the architecture of the tube as depicted by filling the averaged density of tube 2 with a net of atoms (gold) representing the membrane bilayer in turn decorated by scaffolding membrane proteins (blue). These proteo-lipid upright strands are connected by weaker lateral contacts that confer to the highly curved tube wall the fenestrated appearance.

Using PRD1 procapsids we have clarified that the internal pressure due to the packaged DNA does not induce the membrane transformation and consequently both lipids and membrane-associated proteins orchestrate the membrane transition as originally observed in the quantitative biochemical virus dissociation studies [31]. Our data reveal a range of viral membrane shapes (Figures 1B–D, 2A, left, and 3). Particularly striking, membrane morphotypes were the discoid- and stomatoid-like vesicles observed in the procapsids (Figure 3A), mimicking almost the homeostatic functions typical of the plasma membrane of blood cells [38]. This membrane remodelling occurs in response to changes in environmental conditions—namely, osmolarity. By inference in PRD1, the exchange of osmolytes with the external solution through the capsid (*in vitro* vertex de-capping by ageing) (Figure 3A, right) or the direct structural alteration initially caused by the attachment to the cell by the viral receptor binding protein P2 (*in vivo* vertex de-capping) destabilises the icosahedral vesicle, which ultimately leads to the tail tube formation (Figure 7A).

These are universal membrane morphologies that can be modelled by considering the reduction in vesicle volume versus the reduction of monolayer area difference between the two leaflets (area-difference-elasticity theory) [38],[39]. Under specific environmental conditions, vesicles composed only of lipids can also form tubes favoured, for example, by specific lipid compositions [40],[41]. In particular, phosphatidylethanolamine (PE) species lead to negative curvature [42], whereas lipids with negatively charged headgroups respond to changes in pH and/or concentration of ionic strength. Notably, the PRD1 vesicle is composed mainly of PE (53%) and phosphatidylglycerol (PG; 43%), with an asymmetrical distribution of lipids between the two membrane layers with the PE and PG species mainly segregated in the inner and outer leaflet, respectively [9]. However, in PRD1, the transformation of the membrane implies the redistribution of the membrane-associated proteins (occupying ∼50% of the membrane volume) of which only the vertex-stabilizing protein P16 is icosahedrally ordered [8],[24]. This redistribution of the proteins facilitating the tube formation and scission (Figure 3B) is in line with several other model protein-membrane systems [41]–[43].

Thus, considered as a giant unilamellar (proteo-)vesicle, the PRD1 membrane is primed to readily react to environmental changes (Figure 3A, right), rationalising previous observations where, for example, changes in buffer and/or temperature increased tube formation [44].

### Assembly of the Viral Proteo-Lipidic Tunnelling Nanotube

The viral vesicle does not form a hollow cylinder but rather a structured tube (∼4.8 nm thick), implying that the viral membrane-associated proteins act as a scaffold for the tube. Indeed, cross-sectional views for the most ordered tubes (Figure 5C) show alternate regions of high and low density (Figure 5B–C), possibly indicating a multistrand architecture. Also, visual inspection of the two averaged tubes superimposed using the ring-like structure as pivot corroborates that the differences might be variations on a common core assembly (Figure S6).

The observed high-contrast density regions (Figure 5C, insets) may be segregated membrane domains enriched in proteins polymerizing outward from the gate, possibly in an ordered fashion and with a coiling component (Figure 7B–C). The weaker intercalating density could indicate that lateral contacts between the polymerizing building blocks are more labile (Figure 7C, bottom), reflecting the dramatic curvature needed in the proteo-lipidic tube (2r∼8.8 nm; Figure 5D). Candidate scaffolding proteins include the single-pass transmembrane proteins P7/P14 and P32 and the multipass transmembrane protein P18, whose knock-out impairs tube formation [30]. The primary sequences of these do not have any significant similarity with known viral and cellular proteins.

In mature virions, the assembly of the tail tube and its correct direction through the opening of the vertex could be linked to the DNA counterpressure. The limited space in the virion restricts the conformational changes of the vesicle. The putative interaction of ATPase P9 with the viral genome via its terminal protein P8 [20] might serve the nucleation point and guide tail tube polymerization. This is consistent with the fact that in the wt PRD1 the majority of the tubes were rarely seen as short as those detected in the procapsids and confined within the capsid (Figure 3B). Biochemical evidence supports structural crosstalk between the membrane and the unique vertex via the interactions of P22, P20, and P6 in complex with P9 and the packaged viral genome via the terminal protein P8 [19],[20],[22]. Intriguingly, the observed ring-like structure in the two averaged tube volumes matches into the capsid density at the aperture of the de-capped vertex (Figure 7A, inset), suggesting that the ring might be composed of capsid proteins such as peripentonal P3 monomers remaining attached during the tube ejection or proteins specific to the unique vertex.

Finally, the overall geometric parameters of the tail tube—outer diameter ∼14 nm, internal diameter ∼4.5 nm (as from Figure 5D), and average length ∼50 nm—make this the smallest membrane nanotubes known to be capable of transporting biological material. Cellular tunnelling membrane nanotubes (TNTs), such as filipodia, implicated in cell-to-cell bridging and in shuttling different cellular and viral cargos, possess a diameter ranging from 50 to 200 nm [32],[33].

### Model of the Viral DNA Entry Process Via a Tunnelling Nanotube

Our 3D studies of PRD1–cell interactions map *in vivo* the sequence of events leading to infection. The overall *in vitro* tube characteristics are preserved, and in the cellular context, the viral genome delivery device enters almost orthogonally to the cell surface (variance ∼30°). Occasionally, the virus capsid was seen juxtaposed to the cell, producing a detectable infolding of the outer and inner membranes (Figure 6C and Movie S2), with the polymerizing tail tube practically drilling through the entire bacterial cell envelope (Figure 6). Such membrane perforation has also been indirectly followed by measurements of ion gradients across the cell membranes during infection [30], pinpointing proteins P11 and P7 (Figure 1A, right) as the effectors of host cell penetration. In other cases, the virus capsid was seen at a few nanometers from the cell surface, with the assembled tail tube tunnelling through the outer membrane and the cell wall reaching the cytoplasmic membrane (Figure 6A, right, and 6B). The viral tail tube wall does not fuse with the cellular membrane, probably as a result of protein scaffolding protection. The length of the tube, which is on average at least three times longer than the thickness of a typical cell envelope of *S. enterica* (∼15 nm thick), guarantees genome protection during delivery into the cytoplasm (Figure 6A, right, and 6B). Once in the cytoplasmic compartment, release of the viral genomic DNA might be triggered by the intracellular pH conditions that would favour the opening of the distal part (tip) of the tube (black arrow in Figure 1D, top left), allowing the DNA to exit through it, fuelled initially by the energy stored in the pressurized capsid (Figure 7). Additionally, the reactivity of the PRD1 vesicle to environmental changes (Figure 3A) implicates osmotic pressure as a driving force of the genome translocation. The internal diameter of 4.5 nm of the viral nanotube suggests that one double-stranded DNA chain (Ø∼2.6 nm [9]) can be translocated. The internal diameter of this tail tube is in line with that of the proteinaceous tails of the head-tailed bacteriophages. A schematic model summarising the PRD1 infection process is shown in Figure 7A.

### Proteo-Lipidic Nanotubes: “Master Keys” Operating on Different Cellular Locks

Viruses have devised different strategies to protect and to shuttle their genomes into cells. The protruding tail of membrane-containing PRD1 has superficial similarity with the proteinaceous tail of the head-tailed bacteriophages such as T4. However, the origin and nature of the PRD1 nanotube is actually strikingly different.

The PRD1 cell envelope tunnelling mechanism as a novel method of genome translocation is evocative in terms of its proteo-lipidic nature and cargo-shuttling functionality of cellular tunnelling nanotubes used in cell-to-cell communication.

Internal-membrane-containing viruses infect organisms from all cellular domains of life and include bacterial viruses such as PM2 [12], P23-77 [45], and SSIP-1 [46]; archaeal viruses such as SH1 [47], HHIV-2 [48], and STIV [49]; and eukaryotic viruses such as poxviruses, iridoviruses, mimiviruses, and asfarviruses [27],[29],[50],[51], all of which must deliver genetic material into the host cell.

We suggest that the remodeling of the proteo-vesicle into a dynamic membranous tail structure as seen in PRD1 might, suitably adapted to different hosts, underpin a shuttling mechanism common to all such viruses possessing a linear genome.

## Materials and Methods

### Virus Production and Purification

The wt PRD1 and P9-defective mutant *sus1* (for production of procapsids) were propagated in nonsuppressor host *Salmonella enterica* Typhimurium LT2 DS88 and on suppressor strain *Salmonella enterica* Typhimurium LT2 PSA(pLM2), respectively [52]. For wt and procapsid particle production, DS88 cells were infected at an MOI of approximately 8. For procapsid production 15 min after infection, the cells were collected (Sorvall SLA3000 rotor, 5000 rpm, 10 min, 22°C) and resuspended in fresh prewarmed (37°C) growth medium. The particles were purified by polyethylene glycol-NaCl precipitation, rate zonal, and equilibrium centrifugation in sucrose, and concentrated by differential centrifugation (Sorvall T647.5 rotor, 113,580×*g*, 2 h, 5°C) using 20 mM potassium phosphate, pH 7.2, 1 mM MgCl_2_. The protein concentrations were measured by Coomassie blue method using bovine serum albumin as a standard. The specific infectivity of wt specimen was 1–2×10^13^ pfu/mg of protein. Purified procapsids had a low wt/revertant background (titer reduction of 10^4^ on suppressor host PSA and reduction of >10^7^ on nonsuppressor host DS88).

### PRD1 Sample Preparation for Cryo-ET and Cryo-EM

For cryo-ET of individual wt PRD1 and Sus1 particles, a 5 µl volume of 10 nm gold fiducial markers (Aurion BSA gold tracer 10 nm) was mixed with a 10 µl volume of purified PRD1 sample before vitrification process. We applied 4 µl of sample (at ∼0.6 mg/ml) to a 200 mesh R2/1 (or R3.5/1) holey carbon copper grid (Quantifoil Micro Tools GmbH, Jena, Germany) placed in the controlled environment (95% relative humidity) of the Vitrobot (FEI Inc.). After 1 min incubation, the excess of liquid was removed by blotting with filter paper and the grid rapidly plunged into liquid ethane for subsequent data collection. A similar protocol was used for cryo-EM of Sus1 mutants.

For PRD1–cell interaction studies by electron and cryo-electron tomography, DS88 and/or *E. coli* K-12 JE2572(RP4) were used as a host and grown at 37°C. Cells (exponential growth phase, OD_600_ = 0.5) were infected with wt PRD1 at an MOI of 30. At 5 and 30 min p.i., samples were taken and put on ice. The cells were collected by centrifugation (2,000×g, 3 min) and were inserted into sample carrier holders for high-pressure freezing using an EMPACT 2 (Leica). The vitrified samples were freeze-substituted at low temperature using a LFS2 (Leica) as described in [53]. Finally, the resin blocks were sectioned into 200- and 150-nm-thick sections using a 3 mm diamond knife (ultra 45°, Diatome) with an ultramicrotome (UC6, Leica).

For cellular cryo-ET, at ∼30 min p.i. cells were collected by centrifugation (2,000×g, 3 min) and vitrified on quantifoil grids using an automatic plunge freezing apparatus [either a vitrobot (FEI) or a EM GP (Leica)].

### Cryo-ET Data Collection

For PRD1 single particle cryo-electron tomography, vitrified grids were cryo-transferred at liquid nitrogen temperature into a 914 high-tilt tomography cryo-holder (Gatan Inc.) and viewed on a JEOL JEM-2200FS field emission gun (FEG) microscope operated at 200 kV. Tomographic single-axis tilt series of wt and Sus1 particles were collected under low-dose conditions on an UltraScan 4000, 4K×4K Gatan CCD camera (Gatan Inc.), over a tilt range of ±64 with 1.5° increments and at underfocus values ranging from 5 to 8 µm, using the semiautomatic data acquisition software SerialEM [54]. Twenty tilt series at a nominal magnification of 30,000 and a binning factor of 2, thus producing a pixel size of 0.76 nm and 28 tilt series at a nominal magnification of 25,000 and a binning factor of 2, thus producing a pixel size of 0.88 nm, were collected with SerialEM in low-dose mode. The in-column Omega energy filter helped to record images with improved signal-to-noise ratio by zero-loss filtering with an energy window of 30 eV centred at the zero-loss peak. The total dose used for a tilt series was 90–100 electrons/Å^2^.

For epon-embedded PRD1-infected cell studies, tilted series were collected from −60 to +60° at two angles (90° from one another) using a dual-axis tomography holder (2040, Fischione) on a 200 kV FEG microscope (JEOL 2010F) equipped with an Ultrascan 4000 4K×4K camera. For vitrified PRD1-infected cells, a data collection strategy similar to that used for cryo-ET of individual PRD1 particles was adopted.

### Cryo-EM Data Collection of PRD1 Procapsids

Two-dimensional (2D) images were collected on JEOL JEM-2200FS FEG microscope operated at 200 kV at cryogenic temperature and with in-column Omega energy filter, with a 10 eV slit centered at the zero-loss peak. Digital micrographs were recorded under low-dose conditions (∼10 e−/Å^2^ per exposure) with an underfocus range from 2.0 to 6.0 µm at a nominal magnification of 40,000 with an UltraScan 4000, 4K×4K CCD camera (Gatan Inc.), resulting in a final pixel size of 2.8 Å.

### Tomographic Reconstruction of Individual and Cell-Interacting PRD1 Particles

For alignment and 3D reconstruction of the tilted series, we used IMOD and/or Tomo3D software [55],[56]. We used 10 nm gold particles as fiducial markers during alignment, and 3D reconstruction was carried out by weight back-projection and SIRT. No contrast transfer function (CTF) correction was applied, thus limiting our reconstructions to the first zero of the CTF (around ∼1/5 nm in our data-collection setup). Of the several reconstructed tomograms we initially selected 1,207 PRD1 intact particles with a box of 120×120×120 voxels and 251 volumes corresponding to PRD1 particles with a tube and individual tubes, using a box of 140×140×140 voxels.

### Subtomogram Averaging of Intact PRD1 Particles, PRD1 Particles with Tube, Exit-Portal, and Tube Alone

Subtomogram averaging was carried out using *Dynamo* software [35]. The resolution of the different subtomogram averaged maps was assessed by Fourier shell correlation (FSC) between independent half datasets at the 0.5 threshold criterion in *Dynamo* (Figure S2).

In single-particle averaging of intact PRD1 particles, a full range of rotational searches was performed against a PRD1 template model filtered at 8.0 nm with a loose spherical-shell mask including the vesicle and virus spikes (inner and outer radii of 16 and 49 nm). Subsequent refinements of the initial alignment parameters were scaled down to finer search angles and angular intervals but never imposing 60-fold symmetry. A total of 824 subtomograms aligned with a cross-correlation higher than 0.5 of the mean cross-correlation with the reference contributed to the nonicosahedral averaged wt PRD1 structure (Figure S2A).

For nonintact PRD1 particles, the rough orientations of the single subtomograms relative to the tube were clearly recognizable, enabling the construction of a first set of alignment parameters by manual operation on the particles (Protocol S2 and Figure S7A). As a result of this coarse alignment, a crude averaged model filtered to 8 nm was generated and used as a starting template for the global computerized alignment and averaging protocol. Shifts along the tube long axis were limited, whereas a 360° rotation around this particle orientation was searched; a mask inclusive of capsid and tube was used during this process (Figure S7A). The angular search allowed the particle to pivot inside a cone with an aperture of 60° and rotate inside a full range of 360°. An initial angular sampling of 15° was employed in both cases, with three subsequent coarse-to-fine refinement steps that halved the angular interval and operated a new search around the previous cross-correlation maximum. The set of best orientations provided the alignment parameters that generated the reference used in the next iteration. This procedure was iterated four times onto binned particles and the alignment parameters used to compute a constrained covariance matrix of the initial 251 PRD1-tube and tube sub-volumes. The posterior classification by principal component analysis (PCA) using *Dynamo* software [35] allowed reducing the structural heterogeneity of the selected structures to a set of 174 sub-volumes. Then, further steps with finer sampling were carried out onto fully sized particle (pivoting range of 20° and azymuthal rotation range of 20°, in both cases with an initial angular interval of 5°) until no further improvement in the alignment parameters was observed.

Then, different subboxing schemes and masks were used for the single-particle subtomogram averaging of the capsid alone, exit-portal, and tube (Protocol S2).

One hundred and seventy-four particles contributed to the subtomogram averaging of the capsid and 138 to the subtomogram averaging of the exit-gate, whereas 33 and 64, respectively, contributed to the averaged model tube of class 2 and averaged model tube of class 3 (Figure 5C). For the subtomogram averaging of the tubes, an iterative multireference protocol combining alignment and classification was carried out using as initial references four featureless cylindrical shells (Protocol S2). To validate the results of this alignment, a simulated dataset was created and aligned using the same numerical procedure applied onto the real dataset (Protocol S3).

### 2D and Symmetry Analysis of PRD1 Procapsid Tubes

Digitally recorded 2D images of vitrified PRD1 procapsids were normalized and inspected for the presence of tubes lying with the long axis quasi-orthogonally to the projected plane, a nonpreferential orientation as also observed in tomograms. This subset of views of the tube (*n* = 1,758) was extracted using a box with 80×80 pixel dimensions (2.8 Å/pixel). Then, the selected tubes were low-pass filtered to 15 Å and the rotational power spectra calculated with harmonics from 1 to 9 and classified using a 5×5 KerDenSOM classificatory matrix using XMIPP [57].

### Structural Analysis

Extracted cryo-subtomograms used for the analysis of individual PRD1 particles with tube were denoised by anisotropic nonlinear diffusion using TOMOAND software [58]. To minimize possible docking inaccuracy, we used the icosahedral cryo-EM density (EMDB ID 1012) fitted with the PRD1 atomic model (PDB ID 1W8X) as our icosahedral PRD1 reference model. The PRD1 cryo-EM map was then filtered at 6.0 nm resolution to match the resolution achieved with our reconstructions (see the corresponding Fourier shell correlation plots in Figure S2). Using Chimera software [59] “fit-into-map” command, we therefore superimposed our icosahedral PRD1-tube subtomogram averaged capsid density onto the icosahedral cryo-EM capsid map (∼95% correlation; we also checked for coarse magnification errors that are <1.7%). Once the icosahedral version of our cryo-electron tomography reconstruction was oriented onto the PRD1 reference model, we then used it as the target onto which we superimposed our single-particle PRD1-tube averaged map. This allowed the spatial description and localisation of the PRD1 atomic model (PDB ID 1W8X) in the context of our subtomogram averaged densities. *Dynamo*, Chimera, and Amira 5.3.3 (Visage Imaging GmbH, Berlin) software were also used to analyse the averaged maps, to estimate tubes' length and exit angle, and to prepare correspondent figures.

### Accession Codes

#### EM data bank

Subtomogram averaged maps have been deposited under the accession numbers EMD-2437 (gate), EMD-2438 (PRD1-tube; no 60-fold averaged), EMD-2439 (tube average class 2), and EMD-2440 (tube average class 3).

## Supporting Information

Figure S1 Immunogold labelling with antibodies against packaging vertex-associating proteins P20 and P6. (A, Left) Labelling of wt PRD1 with anti-P6 visualized using 10-nm gold with the inset (2× magnification) showing the differential labelling in PRD1 with (T) and without a tube (NT). (A, Right) As left but using anti-P20. The labelling sensitiveness of these antibodies is known to be low [21], however the estimated overall proportions of labelling of NT and T particles (A, Right, Bottom) appear to suggest that there was a difference in the labelling frequencies. (B) Positive control for the labelling procedure carried out with an antibody against major capsid protein P3 (720 copies per virion versus unknown P6 and/or P20 copies per unique vertex), confirming the far more extensive and specific labelling pattern than that shown by anti-P20 and anti-P6. Scale bar, 200 nm for all panels.
(TIF)

Figure S2 wt PRD1 vertices and Fourier shell correlation (FSC) plots. (A, Top) Sections from 1 to 20 (0.88 nm thickness) of the 12 vertices extracted from the single-particle averaged volume of wt PRD1 showing the weak density corresponding to the flexible spike proteins (e.g., white arrow, top left panel). (A, Bottom) FSC between single-particle averaged maps calculated by aligning subtomograms halved in two datasets. The grey line marks the 0.5 threshold criterion for the estimation of the achieved resolution (∼5.8 nm). (B, Top Left) FSC of the non-icosahedral symmetrized PRD1-tube volume calculated as in (A, Bottom). (B, Top Right) As previous but with the averaged gate density. (B, Bottom) As previous with resolution assessment of averaged tube volumes 2 (Left) and 3 (Right).
(TIF)

Figure S3 Tail tube exit from PRD1 procapsids. 2D cryo-image of a PRD1 procapsid sample visualized at 40,000× magnification with particles without and with a protruding tube (Inset) with similar dimensions as those observed for wt PRD1. Scale bar, 30 nm. Black dots, 10 nm nanogold particles.
(TIF)

Figure S4 Clusters from the KerdenSom classificator of harmonics of orthogonal 2D tubes. Self-organizing maps obtained by classification into a kernel density estimator of symmetry spectra calculated by rotational averaging of orthogonal tubes (abscissa, harmonic number; ordinate, relative intensity overall scaled); outlined in red are those clusters showing a clear harmonic 3 and that were used for calculation of the average spectra and image in Figure 5B; outlined in green are those clusters considered with clear 2-fold and marked with 4 to 7 the clusters displaying higher harmonics. Clusters with no labelling were considered spurious.
(TIF)

Figure S5 Experimental and simulated subtomogram tube averaging. (A) Reference models used as initial templates for the multireference procedure. (B) Central sections of the four final averaged classes. (A) and (B) are not to scale. (C) Distribution in the 3D space (the *x*-axis is pointing towards the reader) of the orientations of the tubes' long axis relative to the original extracted box; each line is capped with a coloured dot colour-coded accordingly to the final cross-correlation value (legend) of each tube against the class reference. Depicted with *Dynamo* software. (D, Left) Simulated data according to the initial orientations of members of class 3 (see Protocol S3). (D, Centre) Central sections of the resulting averaged volume using simulated data. (D, Right) Same *z*-slices as Figure 5C but resulting from the simulation, showing that the lobed signature is not replicated during the procedural alignment and averaging protocols.
(TIF)

Figure S6 Superimposition of averaged tubes using the ring-like structure as pivot. (A) Stereo-view of isosurfaces of superimposed tubes (semitransparent gold, averaged tube 2; dark-gold, averaged tube 3) viewed orthogonal to their long axes. (B) Views of tubes as in (A) at different rotation angles. (C, Left) View of the tip end of the tubes. (C, Right) As left but cut-through a plane close to the ring-like structure. Averaged volume 3 was superimposed onto averaged volume 2 using the “dynamo_align” function in *Dynamo* software (cc_i_ = 0.61, cc_f_ = 0.65). After superimposition, volumes were filtered at 5 nm resolution and isosurfaces contoured at 1.2σ in Chimera.
(TIF)

Figure S7 Subtomogram averaging schemes with different masks. (A, Left) Consecutive *z*-slices crossing the center of the initial model. (A, Right) Isosurface representation of the initial model computed by averaging all particles together (*n* = 174) according to the coarse manual alignment. (B, Top) Different masking and averaging schemes focused at different regions of interest: mask C, capsid only; mask CT, capsid and tube; mask T, only tube. (B, Centre) Average density obtained in each case, represented by a gallery of the same *z*-slices chosen in (A, Left); superimposed in fade red on each slice is the extent of the mask used in each case. Below are the corresponding isosurface representations of the averaged densities.
(TIF)

Movie S1 PRD1 infecting *Salmonella enterica* cell (30 min p.i.).
(MP4)

Movie S2 Cell membrane invagination upon PRD1 infection.
(MP4)

Protocol S1 Antibody labelling and negative stain.
(DOC)

Protocol S2 Subtomogram averaging workflow.
(DOC)

Protocol S3 Simulation of tomographic data of a featureless cylinder.
(DOC)

## Abbreviations

EM: electron microscopy
ET: electron tomography
MCP: major capsid protein
