# Supplementary figures and images for: Mechanism of Membranous Tunnelling Nanotube Formation in Viral Genome Delivery
Source: PLoS Biol. 2013 Sep 24;11(9):e1001667. doi: 10.1371/journal.pbio.1001667 (PMC3782422; doi:10.1371/journal.pbio.1001667)

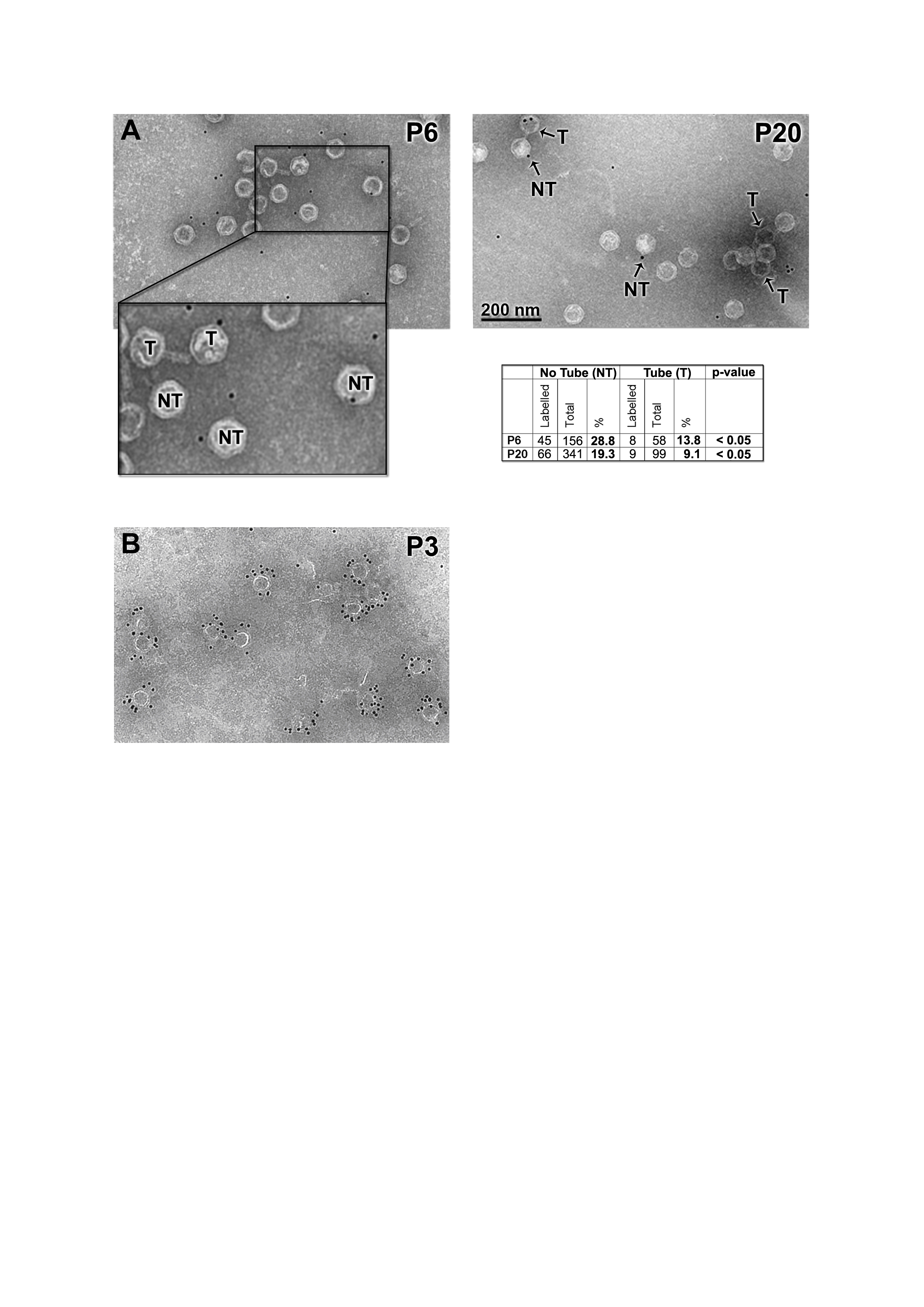

Supplement: Figure S1 — Immunogold labelling with antibodies against packaging vertex-associating proteins P20 and P6. (A, Left) Labelling of wt PRD1 with anti-P6 visualized using 10-nm gold with the inset (2× magnification) showing the differential labelling in PRD1 with (T) and without a tube (NT). (A, Right) As left but using anti-P20. The labelling sensitiveness of these antibodies is known to be low [21], however the estimated overall proportions of labelling of NT and T particles (A, Right, Bottom) appear to suggest that there was a difference in the labelling frequencies. (B) Positive control for the labelling procedure carried out with an antibody against major capsid protein P3 (720 copies per virion versus unknown P6 and/or P20 copies per unique vertex), confirming the far more extensive and specific labelling pattern than that shown by anti-P20 and anti-P6. Scale bar, 200 nm for all panels. (TIF) [file pbio.1001667.s001.tif]

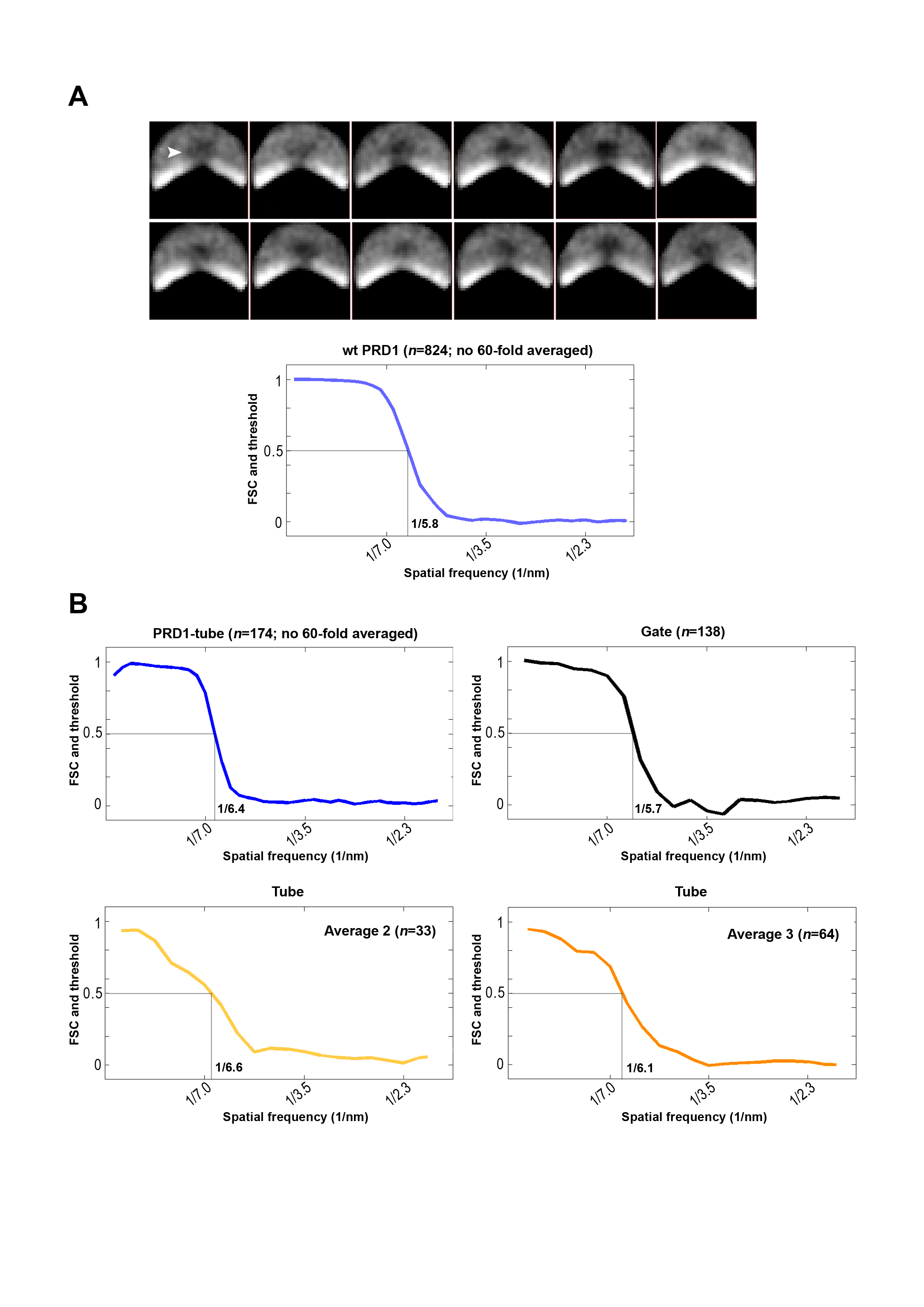

Supplement: Figure S2 — wt PRD1 vertices and Fourier shell correlation (FSC) plots. (A, Top) Sections from 1 to 20 (0.88 nm thickness) of the 12 vertices extracted from the single-particle averaged volume of wt PRD1 showing the weak density corresponding to the flexible spike proteins (e.g., white arrow, top left panel). (A, Bottom) FSC between single-particle averaged maps calculated by aligning subtomograms halved in two datasets. The grey line marks the 0.5 threshold criterion for the estimation of the achieved resolution (∼5.8 nm). (B, Top Left) FSC of the non-icosahedral symmetrized PRD1-tube volume calculated as in (A, Bottom). (B, Top Right) As previous but with the averaged gate density. (B, Bottom) As previous with resolution assessment of averaged tube volumes 2 (Left) and 3 (Right). (TIF) [file pbio.1001667.s002.tif]

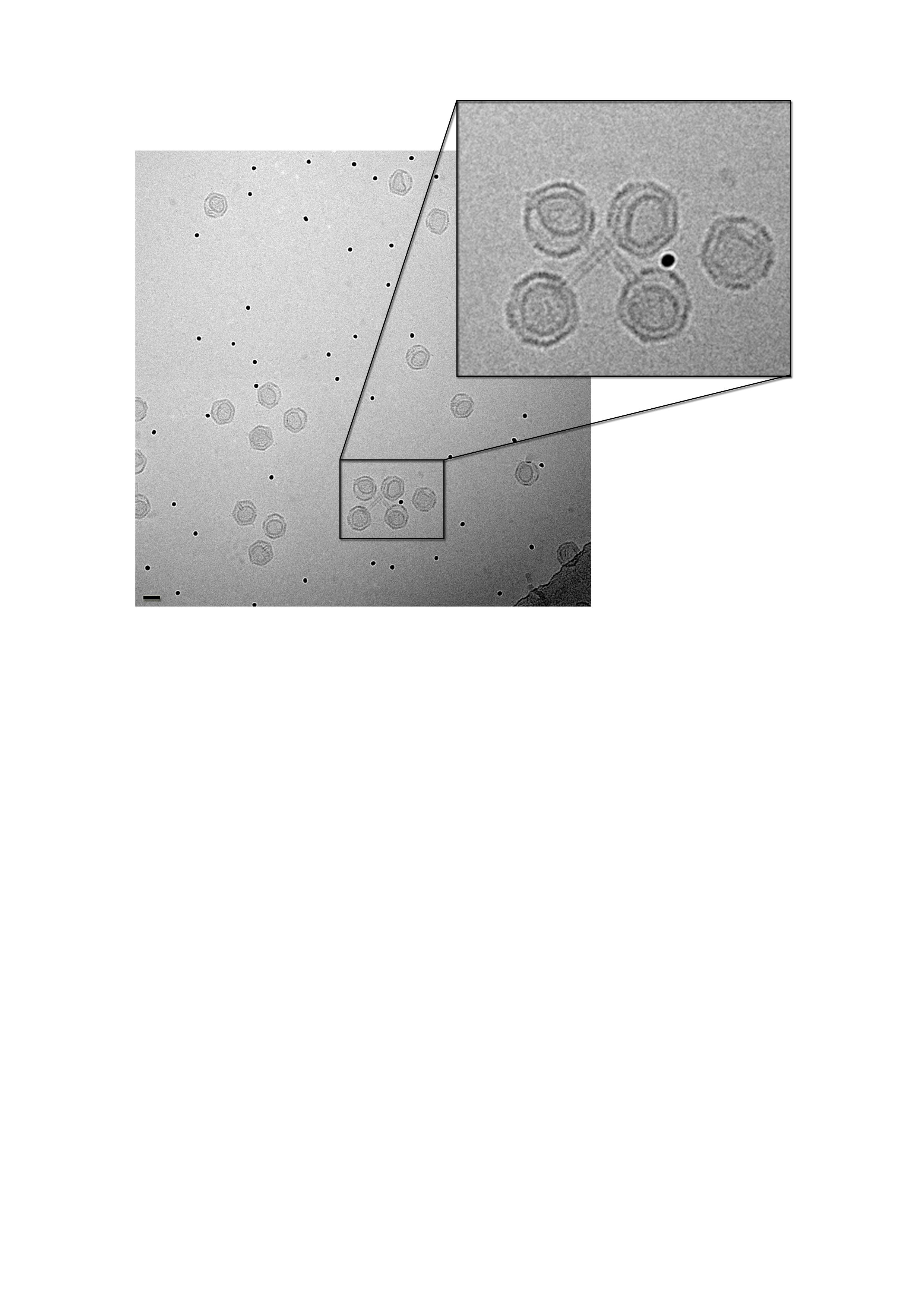

Supplement: Figure S3 — Tail tube exit from PRD1 procapsids. 2D cryo-image of a PRD1 procapsid sample visualized at 40,000× magnification with particles without and with a protruding tube (Inset) with similar dimensions as those observed for wt PRD1. Scale bar, 30 nm. Black dots, 10 nm nanogold particles. (TIF) [file pbio.1001667.s003.tif]

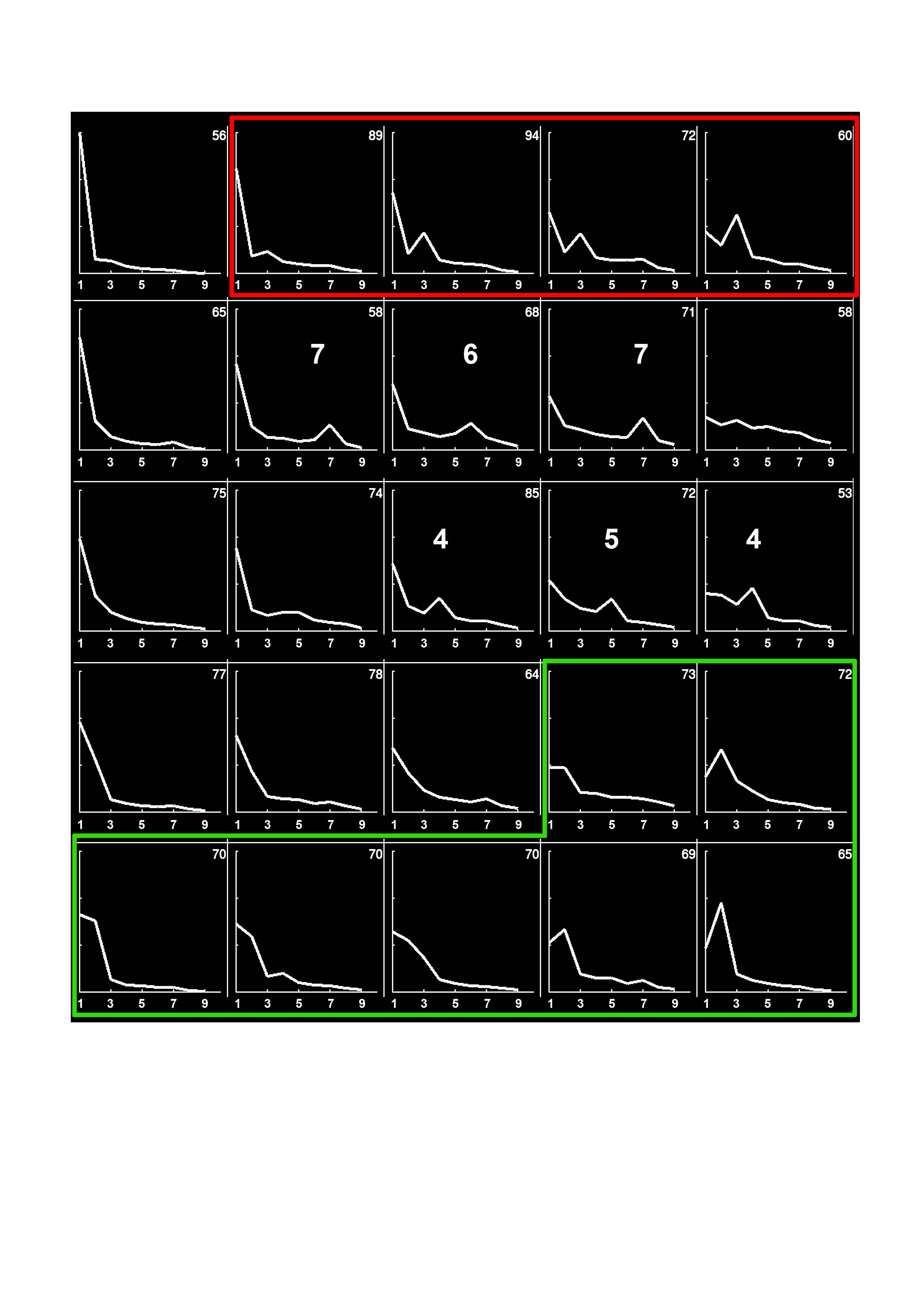

Supplement: Figure S4 — Clusters from the KerdenSom classificator of harmonics of orthogonal 2D tubes. Self-organizing maps obtained by classification into a kernel density estimator of symmetry spectra calculated by rotational averaging of orthogonal tubes (abscissa, harmonic number; ordinate, relative intensity overall scaled); outlined in red are those clusters showing a clear harmonic 3 and that were used for calculation of the average spectra and image in Figure 5B; outlined in green are those clusters considered with clear 2-fold and marked with 4 to 7 the clusters displaying higher harmonics. Clusters with no labelling were considered spurious. (TIF) [file pbio.1001667.s004.tif]

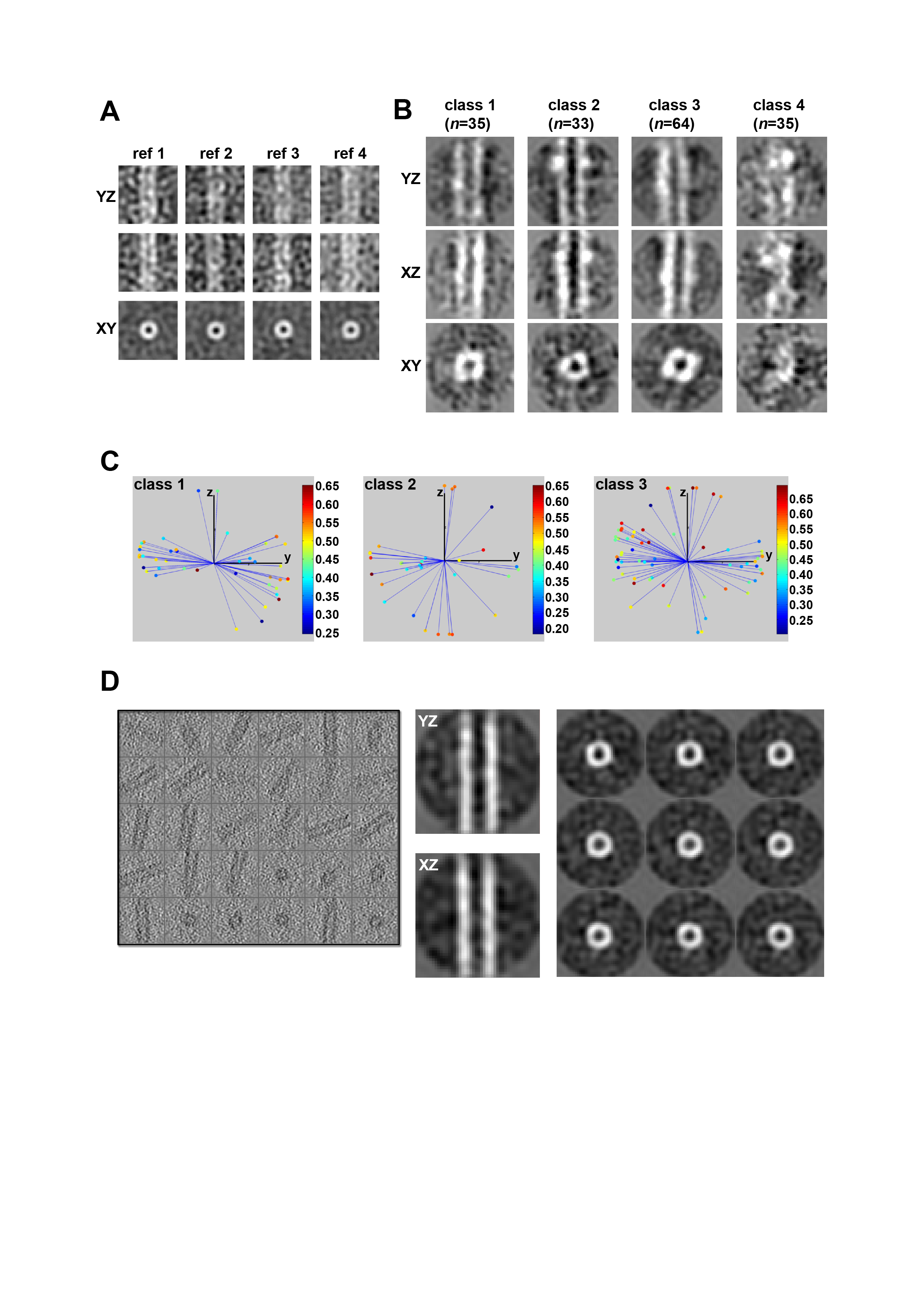

Supplement: Figure S5 — Experimental and simulated subtomogram tube averaging. (A) Reference models used as initial templates for the multireference procedure. (B) Central sections of the four final averaged classes. (A) and (B) are not to scale. (C) Distribution in the 3D space (the x-axis is pointing towards the reader) of the orientations of the tubes' long axis relative to the original extracted box; each line is capped with a coloured dot colour-coded accordingly to the final cross-correlation value (legend) of each tube against the class reference. Depicted with Dynamo software. (D, Left) Simulated data according to the initial orientations of members of class 3 (see Protocol S3). (D, Centre) Central sections of the resulting averaged volume using simulated data. (D, Right) Same z-slices as Figure 5C but resulting from the simulation, showing that the lobed signature is not replicated during the procedural alignment and averaging protocols. (TIF) [file pbio.1001667.s005.tif]

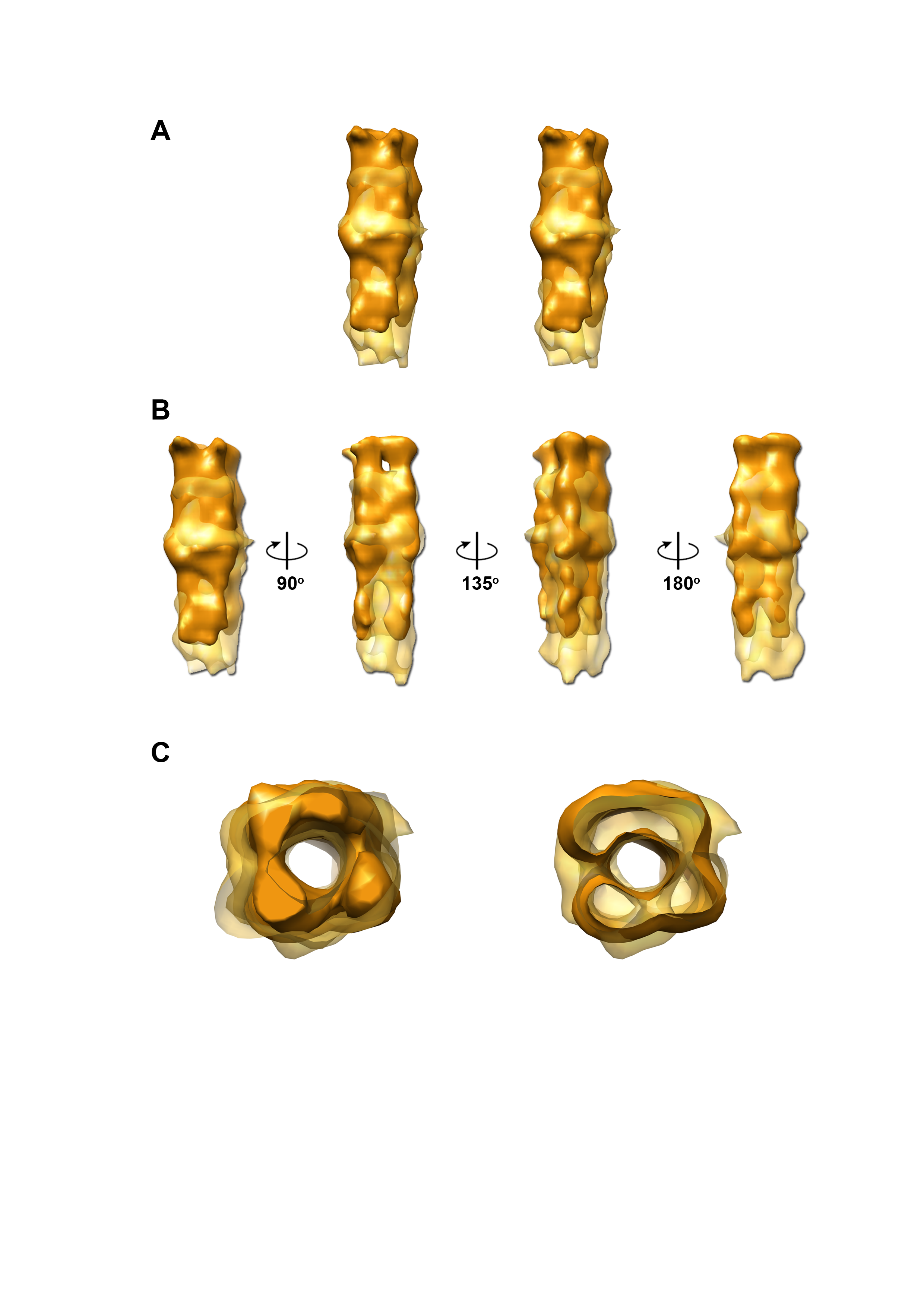

Supplement: Figure S6 — Superimposition of averaged tubes using the ring-like structure as pivot. (A) Stereo-view of isosurfaces of superimposed tubes (semitransparent gold, averaged tube 2; dark-gold, averaged tube 3) viewed orthogonal to their long axes. (B) Views of tubes as in (A) at different rotation angles. (C, Left) View of the tip end of the tubes. (C, Right) As left but cut-through a plane close to the ring-like structure. Averaged volume 3 was superimposed onto averaged volume 2 using the “dynamo_align” function in Dynamo software (cci = 0.61, ccf = 0.65). After superimposition, volumes were filtered at 5 nm resolution and isosurfaces contoured at 1.2σ in Chimera. (TIF) [file pbio.1001667.s006.tif]

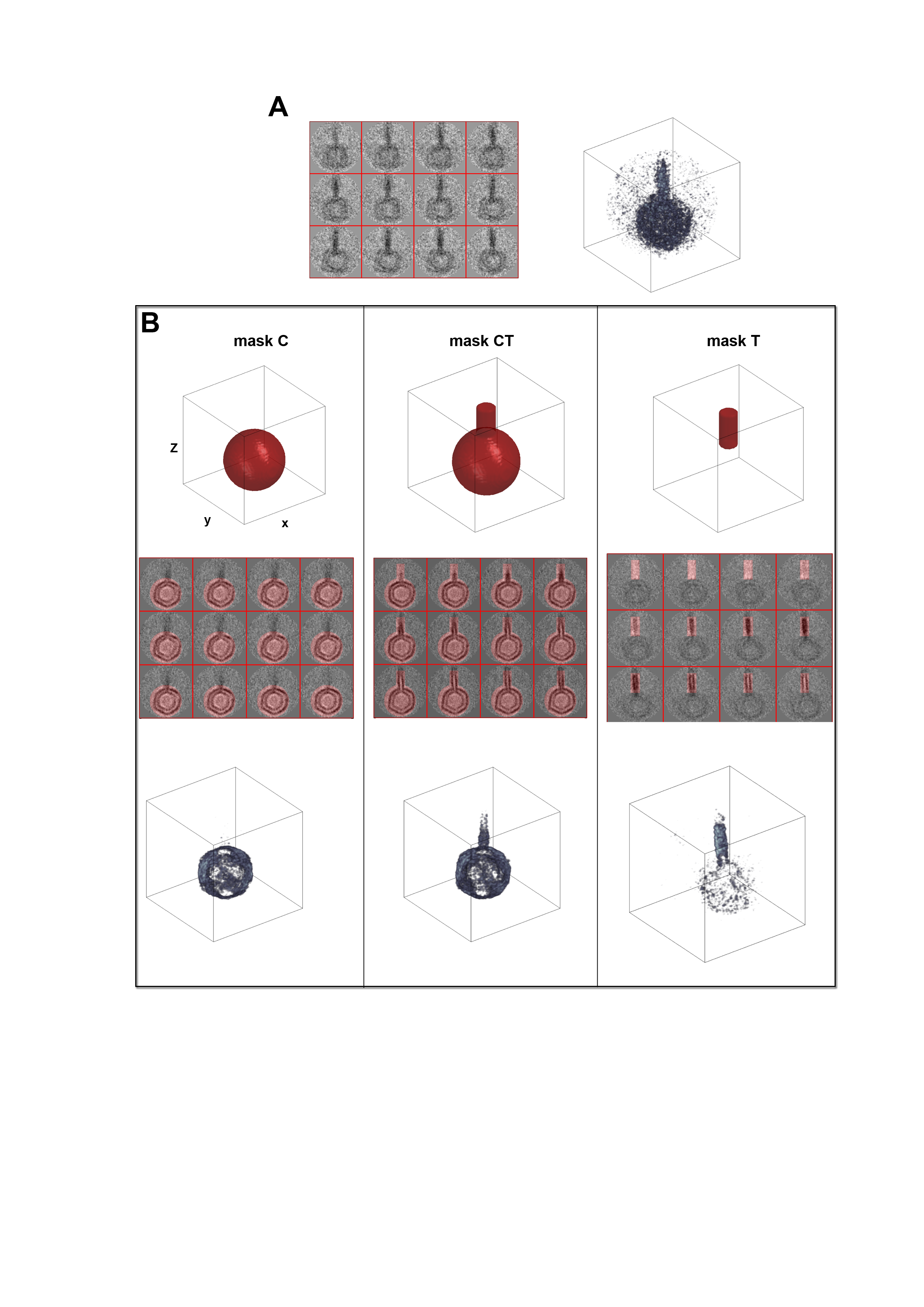

Supplement: Figure S7 — Subtomogram averaging schemes with different masks. (A, Left) Consecutive z-slices crossing the center of the initial model. (A, Right) Isosurface representation of the initial model computed by averaging all particles together (n = 174) according to the coarse manual alignment. (B, Top) Different masking and averaging schemes focused at different regions of interest: mask C, capsid only; mask CT, capsid and tube; mask T, only tube. (B, Centre) Average density obtained in each case, represented by a gallery of the same z-slices chosen in (A, Left); superimposed in fade red on each slice is the extent of the mask used in each case. Below are the corresponding isosurface representations of the averaged densities. (TIF) [file pbio.1001667.s007.tif]
