# Supplementary material for: Mechanism of Membranous Tunnelling Nanotube Formation in Viral Genome Delivery
Source: PLoS Biol. 2013 Sep 24;11(9):e1001667. doi: 10.1371/journal.pbio.1001667 (PMC3782422; doi:10.1371/journal.pbio.1001667)
Supplement: Protocol S1 — Antibody labelling and negative stain. (DOC) [file pbio.1001667.s010.doc]

Specific polyclonal antibodies recognizing PRD1 protein P2 and P20 as well as monoclonal antibody recognizing protein P6 (6A488) were used for labelling [21]. The immuno-labelling protocol of the wt particles was carried out as described [21]. For labelling estimation, images were taken using a JEOL JEM-1230 transmission electron microscope operated at 120 kV under low-dose conditions at room temperature and at a nominal magnification of 25000 by direct recording on a Orius SC1000 CCD camera (Gatan Inc.), with a final pixel size at the specimen of 2.8 Å. Counting of the nanogold-labelling against the unique vertex proteins P6 and P20 was carried out distinguishing between wt PRD1 particles with and without the apparent presence of the attached tube. Images with reasonable nanogold background (Figure S1) were considered whilst those where more than five viruses clumped together as well as particles located at the edges of the image or apparently broken were disregarded. To assess the significance of the differences observed in the proportions of the labelled particles with no tube and with a tube for each of the used antibodies a χ^2^ square test for frequencies comparison was performed.
