# Supplementary material for: Mechanism of Membranous Tunnelling Nanotube Formation in Viral Genome Delivery
Source: PLoS Biol. 2013 Sep 24;11(9):e1001667. doi: 10.1371/journal.pbio.1001667 (PMC3782422; doi:10.1371/journal.pbio.1001667)
Supplement: Protocol S2 — Subtomogram averaging workflow. (DOC) [file pbio.1001667.s011.doc]

Each particle in the data set was visually inspected and two points selected as pivots for the initial manual alignment using *Dynamo* software [35]: the first one in the contact area between capsid and tube and the second at the tip of the tube. Some particles were identified as individual tubes, i.e., tubes not attached to a capsid (Figure 1E). In such cases, the two extremes of the tube were clicked.

Then the sub-tomogram averaging calculations were performed and adapted to three different scenarios described by three following masking schemes: (*i*) mask C, tightly attached to the capsid but excluding the tube, (*ii*) mask CT, inclusive of both capsid and tube and (*iii*) mask T, masking only the tube (Figure S7).

Jointly, the three results of the respective alignments indicate that perfect orthogonality between capsid and tube is not a robust feature in the individual particles: alignment of the tubes (mask T) produces a practical deletion of the capsid in the averaged volume, and alignment of the capsid produces a weakening of the average density corresponding to the tube. CT, in turn, reproduces both features, although the density of the tube is less strong (Figure S7B).

**PRD1 capsid alone alignment and averaging**

Sub-boxes of 96x96x96 voxels were cropped out from each particle and used as data set (*n*=174) for a new subtomogram averaging procedure. A circular mask excluding the tube was used during the alignment (Figure S7). The orientations found during the global alignment were used as starting orientations as well as to generate the initial reference volume. Two independent averaging processes were launched differing in the imposition of icosahedral symmetry at each cycle for one of them. Final results of this procedure are shown in Figure 2. The resolution of the non-icosahedral averaged PRD1 capsid as estimated by the Fourier shell correlation (FSC) to a correlation threshold of 0.5 is 6.4 nm (Figure S2B, top left).

**Gate alignment and averaging**

As for the capsid, sub-boxes of 48x48x48 voxels corresponding to the vertex from where the tube protrudes were cropped out from each particle and used as data set. The alignment parameters found during the global alignment were used as starting orientations and the corresponding volumes averaged to produce the initial gate template.

In the alignment set-up, gate particles were allowed to pivot their axes inside a cone with an aperture of 10^o^, allowing a full range of 360^o^ for azimuthal rotations about the axis. Initial samplings of 5^o^ (pivoting) and 15^o^ (azimuthal rotation) were used to sample the respective ranges, proceeding to a finer search around the best angular positions. This finer search scaled down to angular intervals of 1.25^o^ and 3.75^o^. A tight mask restricting the region of interest was imposed during the alignment procedure. The following final refinement steps were carried out at finer angular searches and sampling rates. A total of 138 gate volumes (with a cross-correlation > 0.4) contributed to the final averaged gate (~5.7 nm resolution; Figure S2B, top right).

**Tube alignment and averaging**

Original tubes possessed variable lengths ranging from ~60 to 40 nm. Sub-boxes of 48x48x48 voxels corresponding to the tube region were extracted from the original PRD1-tube volumes. A multireference alignment was carried out using as starting references four featureless cylindrical shells with dimensions equivalent to the experimental tubes. In each the same level of randomly distributed noise was added and prior the initial alignment round, the four references were filtered to 7.0 nm resolution (Figure S5A). A total of 167 tubes sub-volumes were aligned in this protocol with starting parameters derived by the orientations found in the global alignment. Thus, the multireference alignment was set-up to allow longitudinal and cross-sectional shifts along the tube axis as well as a full 360^o^ azimuthal rotation. During the first five cycles of the alignment procedure shifts were limited within six pixels (~5.3 nm) from the previously found position. Rotation searches were sought inside a pivoting cone of aperture of 30^o^ with full freedom for azimuthal rotation. Two more refinement cycles were performed, constraining pivotal movements in a cone of 7^o^ aperture and azimuthal rotations in a range of 15^o^. Inside a cycle, all particles were compared to each reference. Then, a new set of reference averages were produced by selecting for each reference only those particles that scored their maximum cross-correlation when compared to this reference.

Convergence was reached after 7 cycles and produced the four classes displayed in Figure S5B. Of these four classes (class-1: *n*=35; class-2: *n*=33; class-3: *n*=64; class-4: *n*= 35) class-4 was the visually most structurally incongruous, and also the one showing smaller values of the mean cross-correlation values (respectively 0.45, 0.45, 0.47 and 0.39). The remaining 3 classes were all compatible with a multi-strand architecture although class-2 and class-3 displayed an additional unique ring-like feature (Figure 5C and Figure S5B). Furthermore, analysis of the angular distribution of the set of tubes in class-2 and class-3 showed a reasonable coverage of the geometric sphere, while quality of class 1 was compromised by the low number of tube particles assigned to this class whose original orientation in the tomograms was close to the direction of the electron beam (“orthogonal views”) (Figure S5C).
