# Supplementary material for: Mechanism of Membranous Tunnelling Nanotube Formation in Viral Genome Delivery
Source: PLoS Biol. 2013 Sep 24;11(9):e1001667. doi: 10.1371/journal.pbio.1001667 (PMC3782422; doi:10.1371/journal.pbio.1001667)
Supplement: Protocol S3 — Simulation of tomographic data of a featureless cylinder. (DOC) [file pbio.1001667.s012.doc]

To ascertain that the three-lobed density pattern observed in the averaged tube models was not artifactual a simulated data set was created and analyzed using *Dynamo* software [35]. A featureless cylinder was generated (interior radius of 4 pixels, exterior radius of 8 pixels) and modulated with the nominal CTF of the data set corresponding to a nominal defocus of 8-μm and a voltage of 200 KeV. A copy of the resulting density map was rotated with the alignment parameters found for each of the real particles and the corresponding missing wedges applied. Gaussian noise was added with amplitude arbitrarily chosen to produce a simulated set visually resembling the real data set. The averaging protocol reproduced the featureless cylinder originally employed (Figure S5D). This test infers that the mathematical operations during alignment and averaging do indeed compensate for missing wedge distortions and in turn support the structure detected in some of the tubes.
